# Plasma-Derived Extracellular Vesicles and Non-Extracellular Vesicle Components from APC^Min/+^ Mice Promote Pro-Tumorigenic Activities and Activate Human Colonic Fibroblasts via the NF-κB Signaling Pathway

**DOI:** 10.3390/cells13141195

**Published:** 2024-07-15

**Authors:** Luis A. Arteaga-Blanco, Andrew E. Evans, Dan A. Dixon

**Affiliations:** 1 Department of Molecular Biosciences, University of Kansas, Lawrence, KS 66045, USA; 2 University of Kansas Comprehensive Cancer Center, Kansas City, KS 66103, USA

**Keywords:** colorectal cancer, human colon fibroblasts, extracellular vesicles, inflammation, NF-κB pathway

## Abstract

Colorectal cancer (CRC) is the third most prevalent cancer worldwide. Current studies have demonstrated that tumor-derived extracellular vesicles (EVs) from different cancer cell types modulate the fibroblast microenvironment to contribute to cancer development and progression. Here, we isolated and characterized circulating large EVs (LEVs), small EVs (SEVs) and non-EV entities released in the plasma from wild-type (WT) mice and the APC^Min/+^ CRC mice model. Our results showed that human colon fibroblasts exposed from APC-EVs, but not from WT-EVs, exhibited the phenotypes of cancer-associated fibroblasts (CAFs) through EV-mediated NF-κB pathway activation. Cytokine array analysis on secreted proteins revealed elevated levels of inflammatory cytokine implicated in cancer growth and metastasis. Finally, non-activated cells co-cultured with supernatant from fibroblasts treated with APC-EVs showed increased mRNA expressions of CAFs markers, the ECM, inflammatory cytokines, as well as the expression of genes controlled by NF-κB. Altogether, our work suggests that EVs and non-EV components from APC^Min/+^ mice are endowed with pro-tumorigenic activities and promoted inflammation and a CAF-like state by triggering NF-κB signaling in fibroblasts to support CRC growth and progression. These findings provide insight into the interaction between plasma-derived EVs and human cells and can be used to design new CRC diagnosis and prognosis tools.

## 1. Introduction

Colorectal cancer (CRC) is in the top 10 most prevalent cancers worldwide, affecting both men and women [1,2]. In the US, CRC is one of the most frequently diagnosed cancers with high mortality rates when it is detected at later stages [1,2]. Despite recent advances in the genetics of CRC and the numerous screening programs to reduce CRC incidence, poor treatment, and clinical outcomes highlight the need for a better understanding of the underlying cellular mechanisms related to tumor initiation and progression [3,4]. CRC progression is a multistep process involving genetic and epigenetic changes that influence the tumor microenvironment (TME), which may result in cancer initiation and dissemination [5,6]. The TME is formed by cellular and molecular components, including signaling molecules, cytokines, chemokines, the extracellular matrix (ECM), blood vessels, endothelial cells, immune cells, fibroblasts, and various additional tissue-resident cell types [6]. Fibroblasts generally participate in tissue homeostasis by secreting the ECM of connective tissue to regulate cellular proliferation and differentiation, which are essential for tissue repair after local damage [7]. Fibroblasts in the TME can be educated by tumor cells acquiring an activated state, identified as cancer-associated fibroblasts (CAFs). Among all stromal cells, CAFs are the most abundant stromal components in the TME and have been implicated in modulating CRC dissemination from the early stage of tumor development and metastasis by the secretion of growth factors, cytokines, pro-angiogenic factors, and the ECM [8,9,10]. Previous studies have elucidated that additional secreted factors derived from CAFs, such as extracellular vesicles (EVs), act as the “messengers” for CAFs to crosstalk with tumor cells to facilitate ECM remodeling, immune suppression, and tumor dissemination [11,12,13,14,15]. In this regard, CAFs and CAF-derived EVs play a prominent role in CRC and actively contribute to each cancer stage [7,9]. However, the molecular mechanisms underlying the interactions between tumor-CAF via EV communication in CRC are still complex and have not been fully elucidated. In the past decade, an increasing number of reports have highlighted the importance of studying EVs from cancer cells to understand the molecular pathogenesis of CRC [16]. Due to their ability to act as a molecular messenger, EVs can directly or indirectly modulate the phenotype and function of fibroblasts present in the TME [17,18]. Moreover, tumor cells secrete a large number of EVs from different intracellular origins with pro-tumorigenic activities that are critical for shaping the inflammatory TME to facilitate disease dissemination to distant organs [19,20,21]. EVs play an important role in cellular communication and influence recipient cells by interacting with cell surface receptors or by transferring their contents of bioactive molecules (e.g., cytosolic proteins, nucleic acids, lipids, and signal molecules) upon internalization [22]. Based on sizes and biogenesis, EVs might be classified into two general subtypes: large EVs (LEVs, >200 nm in diameter), derived from the fission of the plasma membrane from apoptotic or healthy cells [23,24], and small EVs (SEV, <150 nm) originating from endosomal or non-endosomal systems [25,26]. Up-to-date investigations describing the molecular and functional characteristics mediated by different EV subsets in the pathogenesis of CRC by modulating colon fibroblast behavior are limited. While it has been demonstrated that tumor cells secrete small EVs that contain specific oncoproteins and RNAs (mRNA, miRNA, non-coding RNA) that can impact tumor metastasis in different cancer types [27,28], information regarding the communication of circulating LEVs and SEVs from APC^Min/+^ mice (a CRC mouse model) on the relationship between tumor cells-CAF and the modulation of CRC progression are poorly explored. Mutations in the *APC* (adenomatous polyposis coli) gene, which results in increased intestinal epithelial cell proliferation and the loss of differentiation, have played essential roles in the tumorigenesis and progression of human colon cancer [29,30]. Here, we used a mouse model of genetic colorectal tumorigenesis, known as the APC^Min/+^ mouse [31,32], in which colorectal tumors are initiated by the loss of the heterozygosity of the APC locus to investigate the relationship between normal fibroblasts and CAFs via EV communication in colon cancer.

Our study aimed to evaluate whether circulating LEVs and SEVs released in the plasma from APC^Min/+^ were able to reprogram normal human colonic fibroblasts into CAF-like cells and to verify the effect of such activation on surrounding cells (not activated fibroblasts). Understanding the mechanisms involved in the crosstalk of tumors and CAFs via extracellular vesicles is critical to comprehending the pathogenesis of CRC and may contribute to designing new strategies that seek to use EVs as candidate biomarkers and therapeutic agents for CRC diagnosis and prognosis.

## 2. Materials and Methods

### 2.1. Animals 

APC^Min/+^ and wild-type (WT) mice in the C57BL/6J background were obtained from The Jackson Laboratory and maintained by breeding APC^Min/+^ males to C57BL/6J females. Mice were bred at the animal care unit (ACU) facility at the University of Kansas and housed in a 12 h light/dark cycle with food and water available ad libitum. Studies were performed in mice at 15 weeks of age, as APC^Min/+^ mice develop >50 tumors throughout their entire intestinal tract at this age [31,32,33]. All mice experiments were performed following the guidelines of the Institutional Animal Care and Use Committees (IACUC).

### 2.2. Plasma Collection and Tissue Preparation for Tumor Counting 

Male or Female 15-week-old WT or APC^Min/+^ mice were exposed to isoflurane (VetOne, Boise, ID, USA). Animals were not involved in any previous procedures nor had they received any drugs. From the mice, 600–700 μL of blood was drawn from a heart puncture using a 25-gauge needle and collected in 1.5 Eppendorf tubes containing an EDTA solution (0.5 M, pH 8). Samples were processed immediately to minimize platelet activation and hemolysis [34,35]. Plasma samples were centrifuged twice at 3000× *g* for 15 min at 20 °C. The supernatant was collected and transferred into a new Eppendorf tube. Approximately 500 μL of plasma were obtained from each blood sample and stored at −80 °C. For tissue preparation, the intestinal tract from WT or APC mice was surgically removed and sectioned into the small intestine (duodenum, jejunum, ilium) and colon. These sections were flushed gently with phosphate-buffered saline (PBS) at room temperature, placed on PBS-soaked absorbent paper, and cut open longitudinally. Tissue samples were fixed overnight in 10% buffered formalin. Tumors in each section were counted under a dissecting scope (Leica MZ6, Buffalo Grove, IL, USA) by two independent observers blinded to the genotype of the mice. 

### 2.3. Cells and Reagents 

The CCD-18Co human colon fibroblasts were purchased from the American Type Culture Collection (ATCC, CRL-1459). CCD-18Co were cultured in Eagle’s Minimum Essential Medium (EMEM) with 10% fetal bovine serum (FBS; HyClone, Logan, UT, USA), 100 U/mL of penicillin and 100 μg/mL of streptomycin (Pen/Strep; ThermoFisher, Waltham, MA, USA). Cells were maintained at 37 °C in a humidified atmosphere with 5% CO_2_.

### 2.4. Extracellular Vesicle Isolation from Mice Plasma Samples

EV subtypes were isolated from 400 µL of plasma as previously described [36], with some modifications. Plasma was centrifuged at 3000× *g* for 15 min to minimize platelets. The resulting plasma samples were diluted 1:6 in ice-cold double-filtered PBS (DF-PBS), filtered through a 0.8 µm pore PES filter (Millipore, Burlington, MA, USA), and then spun at 15,000× *g* for 40 min to pellet large EVs (LEVs, vesicles with a size >200 nm). Pellets enriched with LEVs were washed in PBS and centrifuged at 15,000× *g* for 40 min. The washed pellet was resuspended in DF-PBS. Supernatants from 15,000× *g* centrifugation were filtered through a 0.22 µm pore PES filter (Millipore). Clarified supernatants were subjected to ultracentrifugation at 150,000× *g* for 70 min in a S55S-1186 Swinging Bucket rotor to sediment small EVs (SEVs, vesicles with a size <150 nm). Finally, the pellet-containing SEVs was washed once with DF-PBS at 150,000× *g* for 70 min. The washed pellet was resuspended in DF-PBS. High centrifugation steps (150,000× *g*) were carried out using Sorvall Discovery M120 SE (ThermoFisher). Pellets enriched with LEVs or SEVs were maintained at −80 °C for upcoming assays. Samples were aliquoted to avoid repeated freeze-thaw cycles for characterization and functional studies. We have submitted all relevant data of our experiments to the EV-TRACK knowledgebase (EV-TRACK ID: EV240137) [37].

### 2.5. Nanoparticles Tracking Analysis (NTA)

Pellets enriched with LEVs and SEVs were diluted (1/1000) in DF-PBS before nanoparticle tracking analysis (NTA). Vesicle sizes were measured using a NanoSight LM10 (Malvern Instruments, Malvern, UK) equipped with a 642 nm laser and a high-sensitivity CMOS camera (OrcaFlash2.8, Hamamatsu C11440, NanoSight Ltd., Salisbury, UK). Five videos of 20 s durations were recorded in five replicates per sample with optimized set parameters for LEV and SEV samples. Software parameters included a temperature below 25 °C, a sensitivity of 30–85 frames per second (fps), a shutter speed of 55, and a laser pulse duration equal to that of the shutter duration. After optimization, settings were kept constant between measurements for all the samples. Data were analyzed using the NTA software version 3.4 (Malvern Panalytical, Westborough, MA, USA) and Microsoft Excel 2010 (Microsoft Corp, Redmond, WA, USA). Three independent replicates (*n* = 3) for each EV subtype from WT and APC^Min/+^ mice were analyzed, and the presented results correspond to the meaning of the five videos taken for a given biological sample.

### 2.6. Scanning Electron Microscopy (SEM) 

Samples were prepared as previously described [38], with some modifications. After the last centrifugation, pellets containing LEVs or SEVs were resuspended (50 μL) in 2.5% glutaraldehyde in a cacodylate buffer (0.1 M), with a pH of 7.2, and samples (20 μL) were adhered in glass coverslips, previously covered with Poly-L-lysine (Sigma-Aldrich, St. Louis, MO, USA). After 30 min at 37 °C, coverslips were washed three times in a cacodylate buffer and post-fixed with a solution of 1% OsO_4_, containing 0.8% potassium ferrocyanide and 5 mM of CaCl_2_ for 40 min at room temperature. After new washings, as previously mentioned, the samples were dehydrated in an ethanol-ascending series (50, 70, 90, 100, and 100%) for 5 min for each ethanol concentration. After dehydration, each cover glass was transferred to a coverslip holder (Electron Microscopy Sciences, Hatfield, PA, USA, Cat. #70193-01), immersed in methanol, and placed into a pressure chamber for critical point drying. Samples completed dehydration using a K850 Critical Point Drier (Quorum Technologies Ltd., Lewes, UK), following the procedure suggested by the company. After dehydration, samples remained inside the pressure chamber until imaging. Each cover glass was mounted individually on an aluminum stub with carbon tape and was sputter-coated with 10 nm of gold using an EMS Quorum 150RS sputter coater. Images were acquired on a Field Emission Scanning Electron Microscope (Hitachi High-Tech, Tokyo, Japan, S4700 Type II series; RRID: SCR_020019) with lower and upper secondary electron detectors at a 10.0 kV accelerating voltage, 5–10 uA emission current, aperture 2 (50 µm diameter), an ultra-high resolution operation mode, a condenser lens at 5, and a 5.0 mm working distance. Images were captured at 2560 × 1920 pixels of resolution with an 80 s scan speed and at four different magnifications (15 k, 20 k, 35 k, 40 k, and 50 k), including low magnification for a broader sample view. The critical point drier, the sputter coater, and the SEM analysis were performed at the Microscopy and Analytical Imaging Research Resource Core Laboratory, University of Kansas (RRID: SCR_021801).

### 2.7. Protein Extraction from Cells LEVs and SEVs

To extract cellular proteins, cultured cells were washed twice with DF-PBS and homogenized with a lysis buffer [100 mM of Tris-Cl (pH 6.8), 4% (*w*/*v*) SDS, 0.2% (*w*/*v*) bromophenol blue, 20% (*v*/*v*) glycerol, 200 mM of DTT (dithiothreitol)], and completed with a protease inhibitor cocktail of 1:100 (Sigma-Aldrich) before use. Lysed samples were incubated on ice for 20 min and sonicated with a Branson 250 Digital Sonifier (frequency 30%) three times for 40 s durations and vortexed (1 min) between each cycle to ensure protein homogenization and membrane lysis. Samples were subjected to centrifugation at 10,000× *g* at 4 °C, and the supernatant was boiled at 95 °C for 5 min. For proteins of pellets enriched with EVs, after the final wash step of centrifugation in DF-PBS, LEV and SEV samples were lysed, and proteins were extracted as described above for cell samples. The protein content of cell lysates and EVs was quantified using a Qubit Protein assay (ThermoFisher) and Qubit 4.0 Fluorometer according to the manufacturer’s instructions. 

### 2.8. Western Blotting

The protein profile of fibroblast or EV preparations was carried out as described before with some modifications [38]. The samples were loaded into 10% sodium dodecyl sulfate-polyacrylamide gel electrophoresis (SDS-PAGE) and were run under either reducing or non-reducing conditions, depending on the subsequent use of the primary antibody, before being transferred to Immobilon-FL PVDF Transfer Membranes (Millipore). Membranes were blocked in 5% non-fat dry milk with Tris-buffered saline-Tween 20 (TBS-T, 0.01%) for 1 h at room temperature. Blots were incubated for 18 h at 4 °C with primary antibodies. After washing with TBS-T, the membrane was exposed to secondary antibodies IRDye 680RD goat anti-Rabbit IgG or IRDye^®^ 800CW Donkey anti-Mouse IgG, as required, for 1 h at room temperature, and washed again with TBS-T. The antibodies used for the Western blotting assay, including dilutions and suppliers, are described in Table 1. Protein bands were captured by the Odyssey Fc Imaging System (LI-COR, Lincoln, NE, USA). Relative band intensity was calculated using ImageJ software version 1.53 (NIH, Bethesda, MD, USA). 

### 2.9. CCD-18Co Treatment with Plasma-Derived EVs

CCD-18Co fibroblasts were exposed to pellets enriched with WT-derived LEVs and SEVs (referred to as LEV-WT or SEV-WT) or pellets enriched with APC-derived LEVs and SEVs (referred to as LEV-APC or SEV-APC) at different concentrations ranging from 10 to 70 µg/mL. When indicated, the supernatant of plasma-WT or plasma-APC centrifuged at 3000× *g* for 20 min or the supernatant of plasma-WT or plasma-APC depleted of EVs (referred to as EVFP-WT or EVFP-APC) was added to fibroblasts at the same concentration as the EVs. The EV-free plasma was obtained by ultracentrifugation at 150,000× *g* for 20 h and then filtered through 0.22 μm filters (Merck Millipore, Burlington, MA, USA). Functional studies of fibroblasts treated with plasma-derived EVs are described below.

### 2.10. Wound Healing Assay

The wound recovery assay was carried out as described, with some modifications [39]. CCD-18Co fibroblasts were seeded into 96-well plates (1.5 × 10^4^ cells per well), and 90% confluence was ensured at the time of scratch. One straight scratch wound was made with a 200 µL pipette tip, and non-adherent cells were washed out with PBS. A fresh medium with WT-derived EVs or APC-derived EVs at indicated concentrations (10–70 µg/mL) was added to the wells, and the cells were incubated for up to 72 h. When indicated, EMEM + 1% penicillin/streptomycin + 10 ng/mL rhTGF-β1 (R&D systems, Minneapolis, MN, USA) was used as a positive control. Two technical replicates/conditions were performed. The wound healing was monitored every 24 h until the final time point of three days. At all time points, two areas/wounds were photographed under a bright-field EVOS Fl inverted microscope (AMG) using the 10× objective. The wound closure over time was quantified using ImageJ software (NIH, USA) equipped with a wound healing tool [40].

### 2.11. Cell Proliferation Assay

Cell proliferation was measured using cell counting kit-8 (CCK-8) according to the manufacturer’s instructions (GLPBIO Technology, Montclair, CA, USA). In brief, cells were plated in a 96-well plate at a concentration of 2 × 10^3^ cells per well. Fibroblasts treated with WT-derived EVs or APC-derived EVs at indicated concentrations (10–70 µg/mL) were added to the wells, and the cells were incubated for up to 72 h. When indicated, EMEM + 1% penicillin/streptomycin + 10 ng/mL rhTGF-β1 (R&D systems) was used as a positive control. At each time point, 10 µL of CCK-8 was added into the cell culture medium (100 µL) and incubated at 37 °C for 2 h. The absorbance at 450 nm was measured using a BioTek ELX800 Microplate reader (BioTek, Winooski, VT, USA). The data are representative of five independent experiments and two technical replicates.

### 2.12. Human Cytokine Antibody Array

The Human Cytokine Antibody Array C5 (RayBiotech Inc., Peachtree Corners, GA, USA) was used to determine the relative quantities of 80 cytokines according to the manufacturer’s instructions. The cytokine expression levels were detected in the supernatants of fibroblasts after 72 h of incubation with LEV-WT or LEV-APC and were compared with the untreated control. Arrays were imaged using an Odyssey Fc Imaging System (LI-COR). The signal intensity from membranes was measured using ImageJ software (NIH, USA) equipped with a protein array analyzer tool. Relative protein expression levels were calculated from the mean values of the positive controls.

### 2.13. Preparation of Conditioned Media (CMs)

Conditioned media from fibroblasts treated with APC-derived EVs or left untreated were prepared as previously described, with some modifications [41]. Cells were plated in a 6-well plate at 1 × 10^5^ cells per well. After 24 h of cell culturing, fibroblasts were washed twice with PBS, then exposed to a fresh medium containing LEV-APC or cell media alone for 72 h at 37 °C. The supernatant was collected, centrifuged at 500× *g* for 20 min at room temperature, and filtered (0.22 µm filter) to obtain two conditioned media (CMs). One CM was collected from fibroblasts incubated with a medium alone (CM-CTR), and a second CM was obtained from cells treated with LEV-APC (CM-LEV-APC). The CMs were stored at −80 °C, and repeated freeze-thaw cycles were avoided. New fibroblasts were plated in a 6-well plate after the cells reached 70–80% confluency; they were treated with CM-CTR or CM-LEV-APC in combination with the complete growth medium at a ratio of 1:1 and incubated for 72 h at 37 °C. Fibroblasts left treated with CMs were used as negative controls (CTR). Total mRNAs were extracted from each condition as described below.

### 2.14. RNA Extraction and Real-Time PCR Analyses

Total RNA isolation was carried out using the TRIzol reagent (Invitrogen, Waltham, MA, USA) according to the manufacturer’s instructions. The RNA concentration was determined using the NanoDrop ND-2000 Spectrophotometer (Thermo Scientific). For real-time RT-PCR, 1 µg of total RNA in a 20 µL reverse transcriptase reaction mixture was reverse transcribed to cDNA using the ImProm-II™ Reverse Transcriptase (Promega, Madison, WI, USA) following the manufacturer’s protocols. Quantitative PCR was then carried out in a StepOne Plus Real-Time PCR System (Applied Biosystems, Waltham, MA, USA) using 3 µL of a cDNA template, 800 nM of each primer, and 10 µL of Power SYBR Green PCR Master Mix (Applied Biosystems, ThermoFisher) in a total volume of 20 µL. The primer sequences used are listed in Table 2. The cDNA was amplified with the following program: one cycle at 95 °C for 10 m, followed by 45–50 cycles at 95 °C for 15 s and 60 °C for 1 m, and 1 cycle of a melting curve following cooling at 60 °C for 60 s. The PCR products for each primer pair were subjected to melting-curve analysis to confirm amplification specificity. Relative fold changes in gene expression were calculated using the comparative 2^(−ΔΔCt)^ method.

### 2.15. Statistical Analysis

Statistics were performed using GraphPad Prism software version 10. One-way analysis of variance (ANOVA) was used to compare differences among three or more groups following a normal (parametric) distribution. Dunnett’s post hoc test was used to locate the differences between the groups. The students’ *t*-test was used to compare the means of the two groups. Data are shown as the mean and SD; the differences between values were considered statistically significant when the *p*-value was ≤0.05.

## 3. Results

### 3.1. Characterization of Plasma-Derived EV Subtypes from WT and APC^Min/+^ Mice

**Figure 1:**
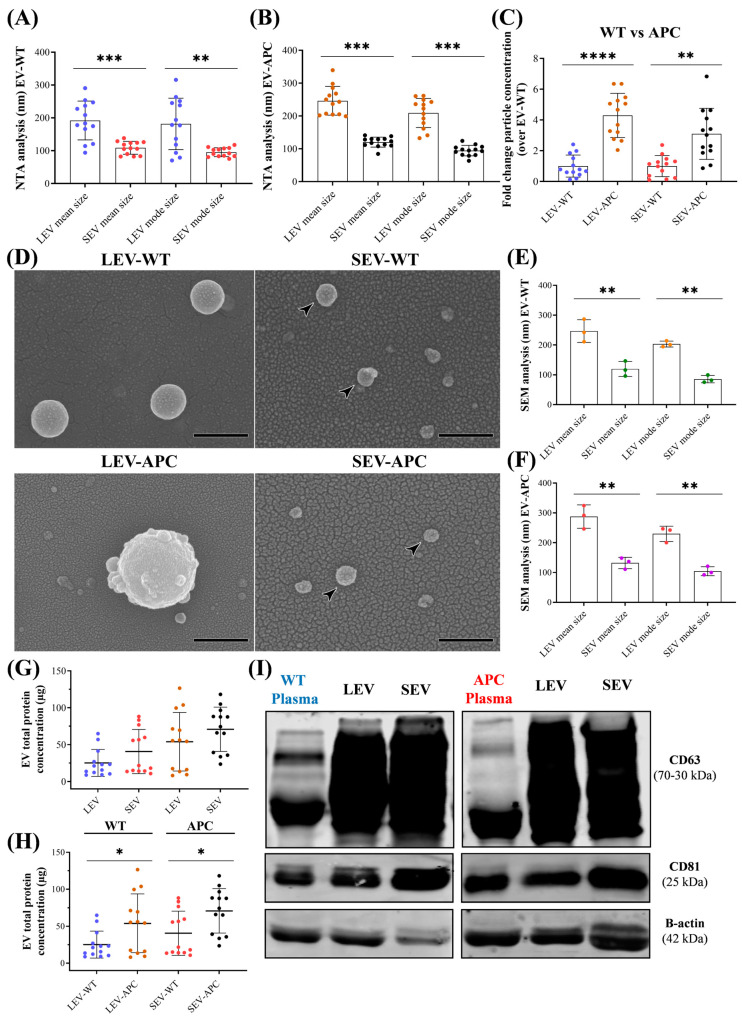
Physical and biochemical characterization of plasma-derived EV subtypes from WT and APC^Min/+^ mice. NTA analysis showing the mean and mode size of pellets enriched with LEVs and SEVs from (**A**) WT and (**B**) APC^Min/+^ mice (*n* = 13). (**C**) Comparisons of particle concentrations of WT-derived EVs and APC-derived EVs (*n* = 13). (**D**) Representative SEM microscopy of pellets enriched with LEVs and SEVs. Images showing homogeneous round-shaped vesicles and other particles with sizes ranging from 50–600 nm (Bars = 400 nm). Black arrowheads point to SEVs with sizes below 150 nm. SEM analysis showing the mean and mode size of LEVs and SEVs from (**E**) WT and (**F**) APC mice via samples from three individual mice (number of images analyzed per mouse = 5). (**G**) Total protein concentrations of EV subtypes (LEVs or SEVs) from WT and APC mice. (**H**) Comparisons of protein concentrations shown in panel G of EV subtypes from normal EVs to EVs released by tumor-bearing mice (*n* = 13). (**I**) Representative blot for EVs markers (CD63 and CD81). β-actin was used as a loading control. * *p* ≤ 0.05, ** *p* ≤ 0.01, *** *p* ≤ 0.001, **** *p* ≤ 0.0001. Abbreviations: LEV: large EVs, SEV: small EVs, NTA: Nanoparticle Tracking Analysis, SEM: scanning electron microscopy.

We began our investigation by removing the intestinal tract from wild-type (WT) mice and the mouse model for CRC, the APC^Min/+^ (Min, multiple intestinal neoplasia) (Supplementary Figure S1A,B). APC mice carry a heterozygous germ-line mutation at codon 850 of the adenomatous polyposis coli (*APC*) gene and exhibit a haploinsufficiency of APC expression [31,32]. The loss of expression of APC in the intestinal epithelium results in tumor formation [33]. At 15 weeks old, APC^Min/+^ mice developed approximately 90 adenomas in the small intestine and 1–4 in the colon (Supplementary Figure S1C). No tumors were present in WT mice. Once we confirmed the two groups of mice in our study, normal and tumor-bearing mice, the blood was collected, and plasma was prepared for EV isolation. EV characterization was performed in compliance with the MISEV 2023 guidelines [42]. Nanoparticle tracking analysis (NTA) showed the enrichment of LEVs from WT and APC mice with a mean size of 190 nm and 240 nm in diameter, respectively (Figure 1A), whereas the SEV preparations showed a mean size value of 108 nm and 120 nm for WT and APC mice, respectively (Figure 1B). SEVs from both mice groups had lower mean and mode size values than LEVs. 

No differences between EV subtypes from WT and APC mice were observed in mean and mode size when evaluated among mice groups (Supplementary Figure S2A,B). Compared with WT mice, plasma-derived EVs from APC mice had a drastic increase in particle concentration, four and three orders of magnitude for LEVs and SEVs, respectively (Figure 1C). The proportion of LEVs recovered with differential centrifugation with a size >151 nm was 70% and 83% for WT and APC mice, respectively (Supplementary Figure S2C). Meanwhile, after the centrifugation steps, 30% and 17% of particles with a size below 150 nm remained in LEVs from WT and APC samples. Similar results were observed in the SEV preparations; the particle yield with a size <150 nm was 86% and 82% for WT and APC mice, respectively (Supplementary Figure S2D). Morphological examination of samples using scanning electron microscopy (SEM) revealed a heterogeneous round-shaped morphology with approximately 246 nm and 290 nm in diameter for LEV samples from normal and tumor-bearing mice, respectively, whereas the SEV preparations showed a mean size of 120 nm and 132 nm for WT and APC mice, respectively (Figure 1D–F). Our analysis was confirmed in EV samples from three mice (Supplementary Figure S3A,B). Size measurements of pellets from three individual mice (number of images analyzed per donor = 5) showed no difference in the mean and mode size between EV subtypes from WT and APC mice (Supplementary Figure S3C,D). According to SEM analysis, we recovered 85% of particles with a size >151 nm for both mice groups in pellets enriched with LEVs. Similarly, SEV preparations for WT and APC samples showed a particle yield with a size <150 nm equal to 86% and 78%, respectively (Supplementary Figure S3E,F). Next, the protein profiles of these particles were compared. We did not initially observe differences in protein concentrations between LEVs and SEVs when associated with mice of the same background (Figure 1G). However, when comparing EV subtypes from WT and APC mice, both EV subtypes released by APC mice had higher total protein contents (Figure 1H). These results suggest that EV subtypes from APC mice showed alterations in particle and protein concentrations as compared with EVs from WT mice. Moreover, immunoblotting analysis confirmed the presence of EV markers (CD63 and CD81) in the preparation lysates (Figure 1I). Altogether, these findings suggest that our protocol allowed the isolation and characterization of EV-containing preparation secreted from normal and tumor-bearing mice by separating pellets enriched with LEVs from preparations enriched with SEVs.

### 3.2. Plasma-Derived EVs Increased Wound Healing in Human Colonic Fibroblasts

**Figure 2:**
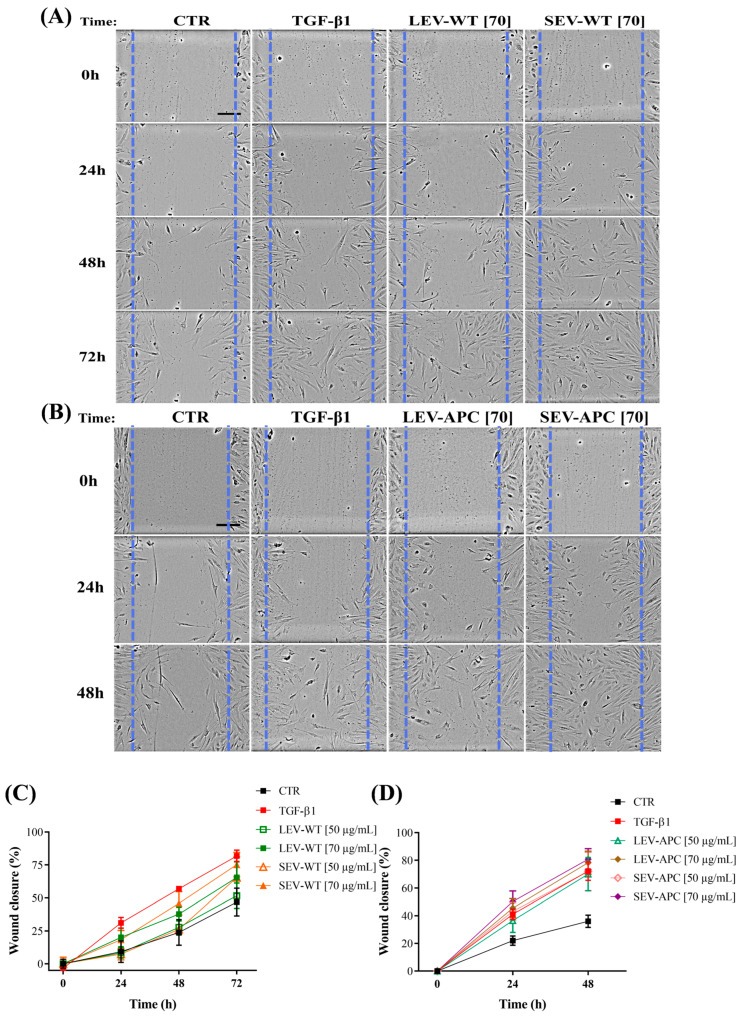
Plasma-derived EV subtypes increased wound recovery in human colonic fibroblasts. CCD-18Co fibroblasts were cultured for 24 h, and a scratch was made using a pipette tip, then cells were exposed to 50 µg/mL or 70 µg/mL of pellets enriched with LEVs or SEVs from (**A**) WT and (**B**) APC mice (*n* = 4). Recombinant human TGF-β1 (10 ng/mL) was used as a positive control. (**A**,**B**) Representative micrographs of all conditions at time points of 0 h, 24 h, 48 h, and 72 h (Bars = 200 μm). Blue dotted lines represent the size of the original wound. All images were acquired using a 10× objective. Graphs at the bottom show the percentage of the wounded area in fibroblasts treated with (**C**) WT-derived EVs and (**D**) APC-derived EVs at indicated time points to the original wound (conventionally set as 100%). Data are shown as means ± SD and represent four independent experiments with two technical replicates (*n* = 4). Statistical differences for each condition are presented in the supplementary figure (Supplementary Figure S4).

Previous studies have demonstrated that CRC cell-derived small EVs promoted cell migration in normal human fibroblasts [43]. We hypothesized that both EV subtypes, LEVs and SEVs, released in the plasma from APC mice, have the same capability of tumor-derived SEVs to induce cell migration. To this end, we treated CCD-18Co human fibroblasts with 50 and 70 µg/mL of EVs isolated from WT or APC mice and assayed the wound repair by evaluating the percentage of wound closure over 24 h, 48 h, and 72 h. As shown in Figure 2, at 72 h, the migration area significantly increased in cells exposed to EVs from both mice groups when compared with untreated cells. Except for the SEV-WT (70 µg/mL) treatment at 48 h, wound recovery was not observed in fibroblasts exposed to LEV-WT and SEV-WT at any concentration tested after 24 h and 48 h of treatment (Figure 2, Supplementary Figure S4A,B). In contrast, APC-derived LEVs at 70 µg/mL closed scratch wounds, achieving 45% and 75% wound recovery at 24 h and 48 h, respectively, compared to 22% and 35% of control cells kept only in media for the same period (Supplementary Figure S4D). Similarly, the extent of the wound closure of fibroblasts exposed to SEVs from the same mice group at 24 h and 48 h was 50% and 80%, respectively (Supplementary Figure S4E). Wound healing was also detected in cells treated with lower concentrations of both EV subtypes from APC mice, except LEV-APC at 24 h (Supplementary Figure S4D,E). No difference was observed in the biological activities mediated by LEVs or SEVs from APC mice. Recombinant TGF-β1 at 10 nM ng/mL, used as a positive control, significantly enhanced wound repair compared with untreated cells at indicated time points (Supplementary Figure S4C,F). These results demonstrated that EVs derived from tumor-bearing mice promoted wound recovery in human colon fibroblasts in a time- and dose-dependent manner.

It has been demonstrated that the functional activity mediated by EVs may be attributed to the EVs alone or to the combination of EVs with other non-EV components, including extracellular particles (EPs) and non-vesicular extracellular particles (NVEPs), which are co-isolated with the EVs during centrifugation steps [24,42]. To determine whether the wound healing effect observed was due only to the EV-enriched preparations or other soluble factors, fibroblasts were exposed to a plasma-WT, plasma-APC, or EV depleted from the plasma of WT or APC mice (referred to as EVFP, as for EV-free plasma). Depleting particles by ultracentrifugation at 150,000× *g* for 20 h reduced 73% and 67% of the particle yield in plasma from WT and APC mice, respectively, compared with the original samples (Supplementary Figure S5). Once the degree of particle reduction was reported. We found that plasma from WT or APC mice significantly increased the migration capacity compared to cells alone in media at all time points (Supplementary Figure S6A,C). However, fibroblasts exposed to EVFPs from both mice groups had no effect on wound repair (Supplementary Figure S6B,D). These findings suggest that the presence of EVs in the EV-containing preparations from tumor-bearing mice is essential to promote fibroblast migration in a time-dependent manner. In addition, based on our dose-response analysis, we performed the subsequent in vitro experiments using 70 µg/mL of protein concentrations as inputs of LEVs or SEVs.

### 3.3. Plasma-Derived EVs Increased Cell Proliferation in Colon Fibroblasts

**Figure 3:**
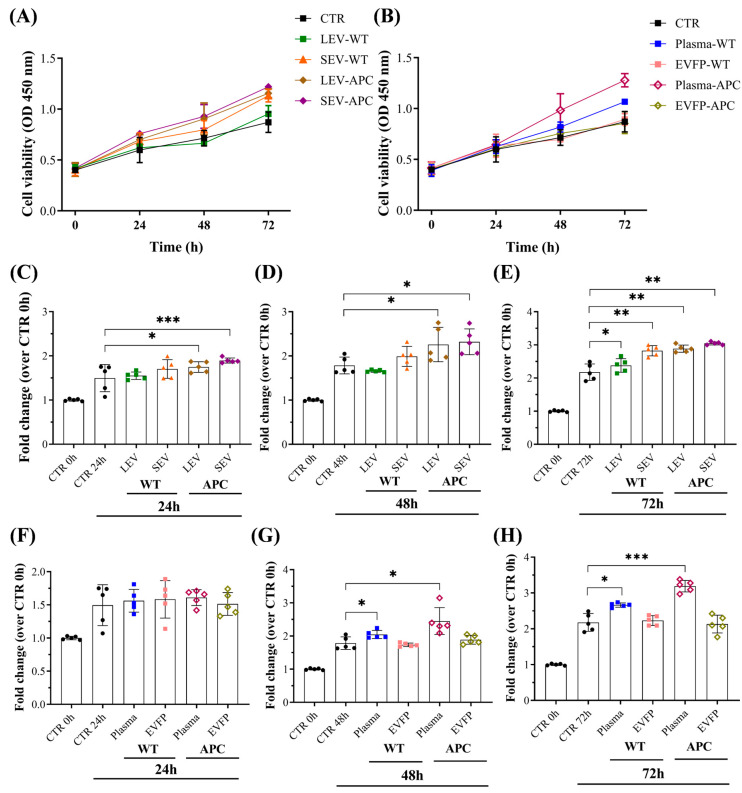
Plasma and EVs promoted cell proliferation in fibroblasts. CCD-18Co cells were cultured for 24 h and then exposed to WT-derived EVs, APC-derived EVs, plasma-WT, plasma-APC, EVFP-WT, or EVFP-APC at 70 µg/mL. (**A**,**B**) The cell proliferation with all conditions was monitored every 24 h until the final time point of three days with an OD value at 450 nm using a CCK-8 kit. The bar graphs show cell proliferation at (**C**,**F**) 24 h, (**D**,**G**) 48 h, and (**E**,**H**) 78 h after the indicated treatments. Data are shown as means ± SD and represent five independent experiments with two technical replicates (*n* = 5). Fold changes were determined over normalized cells kept only with a culture medium (CTR). * *p* ≤ 0.05, ** *p* ≤ 0.01, *** *p* ≤ 0.001. One-way ANOVA followed by Dunnett’s multiple comparisons post-test. Abbreviations: EVFP: EV-free plasma.

To address whether EV preparations may have stimulated cell proliferation in addition to cell migration during wound closure, fibroblasts were treated with WT- or APC-derived EVs, and cell proliferation was performed using a CCK-8 assay (Figure 3). Increased proliferation, approximately 0.25- and 0.4-fold, was detected in the fibroblast culture on day one after treatment with LEVs and SEVs from APC mice, respectively, compared with untreated cells. The proliferation rates significantly increased on days 2 and 3 (Figure 3C–E). In contrast, LEVs and SEVs from WT mice only promoted cell proliferation after 3 days of treatment. The fibroblast proliferation was further analyzed in cells exposed to plasma or EVFPs from WT or APC mice. We found that either WT- or APC-derived plasma elevated cell growth significantly compared to control cells, while no alterations in the rates were observed for EVFP treatments (Figure 3F–H). These results demonstrate that tumor-bearing derived EVs have a stronger capacity to promote fibroblast proliferation in a time-dependent manner, and the presence of EV subtypes in the preparations is critical for cell growth stimulation.

### 3.4. Plasma-Derived EVs from Tumor-Bearing Mice Increased Extracellular Matrix (ECM) Components in Fibroblasts

**Figure 4:**
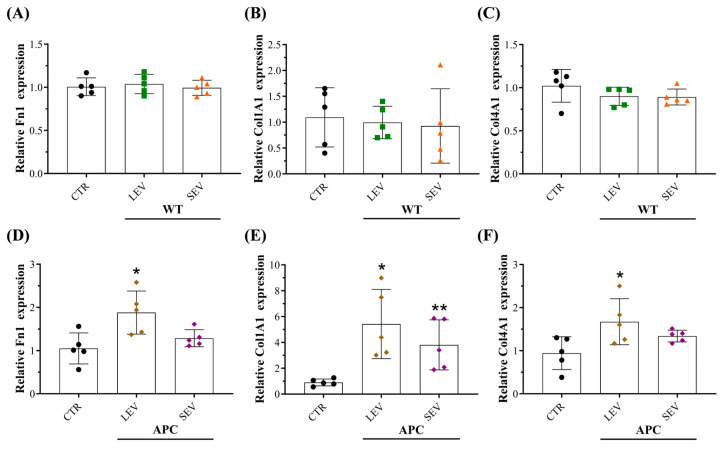
Plasma-derived EVs from tumor-bearing mice increased extracellular matrix (ECM) components in fibroblasts. Relative expression levels of ECM components Fn1, Col1A1, and Col4A1 in fibroblasts exposed to (**A**–**C**) WT-derived EVs or (**D**–**F**) APC-derived EVs. The RNA expression levels were evaluated by qPCR 72 h post-treatment with EV subtypes. Data are shown as means ± SD and represent five independent experiments (*n* = 5). Fold changes were determined over normalized cells kept only with culture medium (CTR). Gene expression was normalized to the housekeeping gene β-actin. * *p* ≤ 0.05, ** *p* ≤ 0.01. One-way ANOVA followed by Dunnett’s multiple comparisons post-test. Abbreviations: ECM: extracellular matrix, Fn1: Fibronectin 1, Col1A1: Collagen type I alpha 1 chain, Col4A1: Collagen type IV alpha 1 chain.

Evidence has demonstrated that fibroblasts are the primary cell types in stromal extracellular matrix (ECM) production and remodeling [44,45]. To gain more insight into the biological function of APC-derived EVs in CRC, the expression of some ECM components, including fibronectin I (Fn1), collagen type I (Col1A1), and collagen type IV (Col4A1), was evaluated in the total mRNA from the fibroblasts after 72 h of treatment with EV-containing preparations (Figure 4). We found that fibroblasts exposed to LEV-APC resulted in a significative upregulation of Fn1, Col1A1, and Col4A1, achieving a 1.9-, 5.4-, and 1.7-fold change, respectively, when compared with untreated cells. Moreover, the SEV-APC treatment increased the Col1A mRNA levels by about 3.8 levels of magnitude by having no effect on the expression of Fn1 and Col4A1 (Figure 4D–F). None of the EV preparations from WT mice influenced the expression of the analyzed ECM components (Figure 4A–C). These findings suggest that tumor-bearing derived EVs increase fibroblasts’ fibronectin and collagen mRNA levels to enhance the secretion of the ECM that might participate in tumor growth.

### 3.5. Plasma-Derived EVs from Tumor-Bearing Mice Reprogram Normal Colon Fibroblasts into CAFs

**Figure 5:**
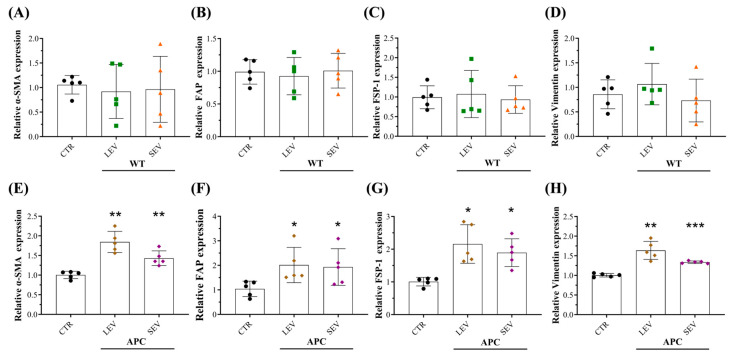
Plasma-derived EVs from tumor-bearing mice increased cancer-associated fibroblast (CAF) RNA levels in CCD-18Co. Relative expression levels of CAF markers including α-SMA, FAP, FSP-1, and Vimentin in fibroblasts exposed to (**A**–**D**) WT-derived EVs or (**E**–**H**) APC-derived EVs. The RNA expression levels were evaluated by qPCR 72 h post-treatment with EV subtypes. Data are shown as means ± SD and represent five independent experiments (*n* = 5). Fold changes were determined over normalized cells kept only with a culture medium (CTR). Gene expression was normalized to the housekeeping gene β-actin. * *p* ≤ 0.05, ** *p* ≤ 0.01, *** *p* ≤ 0.001. One-way ANOVA followed by Dunnett’s multiple comparisons post-test. Abbreviations: CAF: cancer-associated fibroblast, α-SMA: Alpha smooth muscle actin, FAP: Fibroblast activation protein, FSP-1: Fibroblast-specific protein 1.

It has been reported that tumor-derived EVs remodel the stromal ECM by reprogramming normal fibroblasts (NFs) into cancer-associated fibroblasts (CAFs) [17,46,47]. Based on our preliminary results showing that EV preparations enriched with LEVs or SEVs from the CRC mouse model increased the mRNA levels of ECM in fibroblasts, we hypothesized that APC-derived EVs activate NFs into CAFs to enhance ECM secretion. To test this, colon fibroblasts (CCD-18Co) were treated with EV preparations isolated from WT or APC mice. After 72 h of incubation, total mRNA and protein were extracted, and the expression of some fibroblast-activation markers such as α-smooth muscle actin (α-SMA), fibroblast activation protein (FAP), fibroblast-specific protein 1 (FSP-1) and vimentin were evaluated. qRT-PCR and Western blot analysis showed that CAF markers were markedly increased in fibroblasts with APC-derived EV subtype additions (Figure 5, Supplementary Figure S7), except for vimentin in cells exposed to LEV-APC (Supplementary Figure S7). No difference in mRNA or protein levels was observed between control and EV subtypes from WT mice. Blotting membranes for CAF markers are also shown in Supplementary Figure S8. Taken together, our findings indicate that tumor-bearing derived EV subtypes are endowed with the ability to reprogram colon fibroblasts into CAFs. 

### 3.6. Tumor-Bearing Mice-Derived EVs Induced Pro-Inflammatory Responses in Colon Fibroblasts

**Figure 6:**
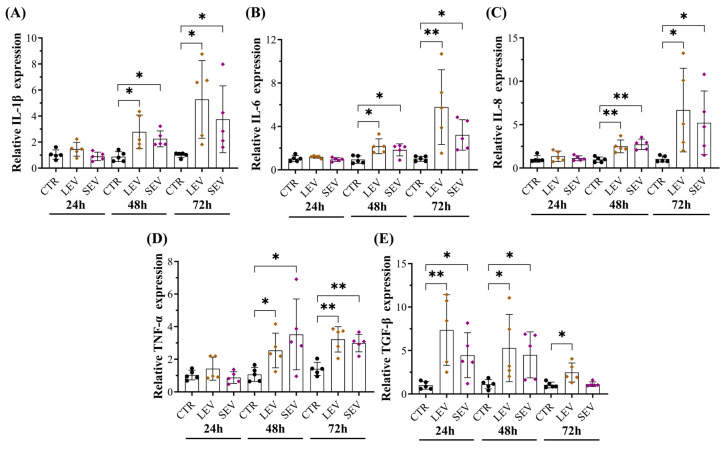
Plasma-derived EVs from tumor-bearing mice increased RNA levels of pro-inflammatory cytokines in fibroblasts. Relative expression levels of (**A**) IL-1β, (**B**) IL-6, (**C**) IL-8, (**D**) TNF-α, and (**E**) TGF-β in fibroblasts exposed to pellet enriched with LEVs and SEVs from APC^Min/+^ mice. The RNA expression levels were evaluated by qPCR in cells at 24 h, 48 h, and 72 h. Data are shown as means ± SD and represent five independent experiments (*n* = 5). Fold changes were determined over normalized cells kept only with a culture medium (CTR). Gene expression was normalized to the housekeeping gene β-actin. * *p* ≤ 0.05, ** *p* ≤ 0.01. One-way ANOVA followed by Dunnett’s multiple comparisons post-test. Abbreviations: TNF-α: Tumor necrosis factor alpha, TGF-β: Transforming growth factor beta.

Multiple factors have been involved in activating normal fibroblast into CAFs, including DNA damage, TGF-β, cellular stress, and inflammatory mediators from paracrine and autocrine signaling [48]. In addition, recent studies have demonstrated that tumor-derived EVs reprogram the CAF phenotype by increasing inflammation and molecular signatures related to cancer progression [47,49,50]. We next asked whether APC-derived EVs can activate pro-inflammatory signaling in colon fibroblasts. qPCR analysis revealed that CCD-18Co cells incubated with EVs or plasma from APC mice significantly increased the expression of multiple cytokines such as IL-1β, IL-6, IL-8, TNF-α, and TGF-β (Figure 6, Supplementary Figure S9A–E), while the upregulation effects were efficiently suppressed after treatment with EV-depleted samples (Supplementary Figure S9F–J). Cytokine expression levels in cells exposed to WT-derived EVs remained unchanged (Supplementary Figure S10). These findings suggest that EV subtypes from tumor-bearing mice induced pro-inflammatory responses in colon fibroblasts in a time-dependent manner, and such inflammation microenvironments could contribute to reprogramming NFs into an inflammatory CAF phenotype.

### 3.7. APC-Derived EVs Increased Soluble Mediators in Fibroblasts Involved in CAF Transformation and Cancer Progression

**Figure 7:**
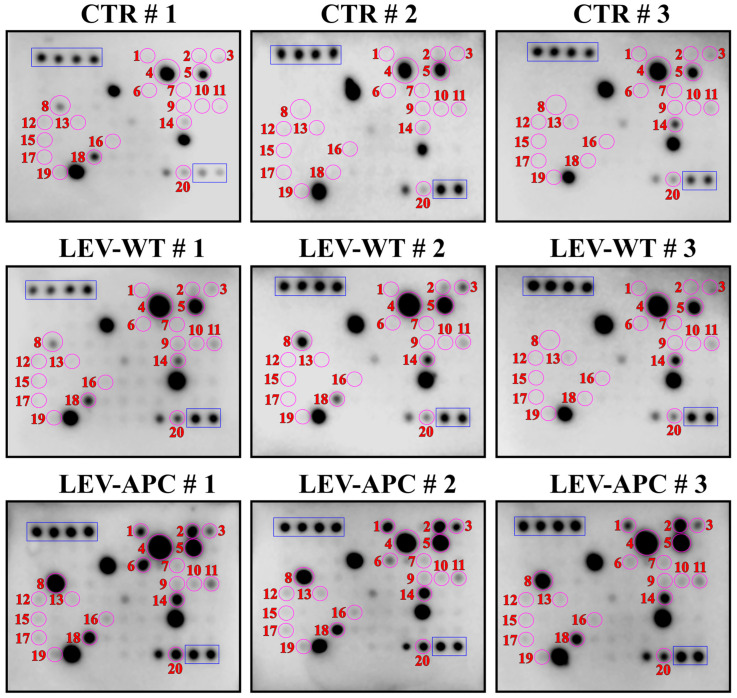
Human cytokine antibody array profiles. Original antibody arrays corresponding to Figure 7. The human cytokine antibody array (*n* = 3) was used to probe cytokine levels in the supernatants of untreated (CTR) fibroblasts, cells treated with LEV-WT, or treated with LEV-APC (70 µg/mL). The expression levels were detected after 72 h of incubation with LEV-WT, LEV-APC, or untreated cells. The circles in purple and numbers in red indicate the spots corresponding to 1 = ENA78 (CXCL-5), 2 = GRO (alpha/beta/gamma), 3 = GRO alpha (CXCL1), 4 = IL-6, 5 = IL-8, 6 = CCL7, 7 = CCL22, 8 = Rantes (CCL5), 9 = EGF, 10 = IGF-1, 11 = Angiogenin, 12 = OSM, 13 = VEGF-A, 14 = CCL11, 15 = FGF-4, 16 = FLT-3 ligand, 17 = IGFBP-3, 18 = CXCL-10, 19 = Osteopontin, 20 = TIMP-2. The positive controls for the membranes are indicated in the blue rectangles. Abbreviations: CTR: control, ENA78: Epithelial Neutrophil-Activating Peptide 78, GRO: The CXC chemokines growth-regulated oncogene (GRO) alpha, GRObeta, GROgamma, CXCL1: chemokine (C-X-C motif) ligand 1, IL-6: Interleukin-6, IL-8: Interleukin-8, CCL7:C-C motif chemokine ligand 7, CCL22: C-C motif chemokine 22, CCL5: C-C motif chemokine 5, EGF: Epidermal growth factor, IGF-1:Insulin-like growth factor 1, OSM: Oncostatin M, VEGF-A: Vascular endothelial growth factor A, CCL11:C-C motif chemokine 11, FGF-4: Fibroblast growth factor 4, FLT-3 ligand: FMS-like tyrosine kinase 3 ligand, IGFBP-3: Insulin-like growth factor binding protein 3, CXCL-10: C-X-C motif chemokine ligand 10, TIMP-2: Tissue inhibitor of metalloproteinases 2.

Tumor development, progression, recurrence, and metastasis can be regulated by alterations in major components of the TME, including the conversion of NFs into a CAF phenotype, which leads to an increase in the aggressiveness of the tumor cells [13,48]. In addition, bioactive molecules carried by EVs can promote neoplastic transformation in CAFs, which are widely involved in the different stages of cancer development and progression [51,52,53]. We next investigated whether APC-derived EVs can stimulate soluble factors in colon fibroblasts involving CAF activation and cancer tumorigenesis. To this end, the supernatants of cultivated CCD-18Co with LEVs from WT or APC mice after 72 h were analyzed for cytokine profiling. Analysis of soluble factors at the protein level by utilizing a cytokine array revealed that LEVs from APC mice, but not from WT mice, upregulated the expression of 20 cytokines and chemokines including IL-6, IL-8, CCL11, CCL22, EGF, angiogenin, OSM, VEGF-A, FGF-4, FLT-3 ligand, IGFBP-3, CXCL-10, osteopontin and TIMP-2 (Figure 7, Supplementary Figure S11, Table 3 and Table 4) more than 1.5 times. We also showed that 3 of those 20 cytokines were found to be >5.5 times more expressed in cells exposed to LEV-APC than untreated fibroblasts: the CXC chemokines growth-regulated oncogene alpha (GRO-Alpha), C-C motif chemokine 5 (CCL5), and the Insulin-like growth factor 1 (IGF-1). Moreover, three other cytokines of those 20 were markedly increased (more than 20 times) in colon cells with APC-derived LEV additions, including Epithelial Neutrophil-Activating Peptide 78 (ENA78 or CXCL-5), the CXC chemokines growth-regulated oncogene (GRO), and the C-C motif chemokine ligand 7 (CCL7) (Supplementary Figure S11, Table 3). These secreted proteins can induce numerous hallmarks of cancer, including angiogenesis, cell proliferation, immune cell infiltration, tumor growth, migration, invasion, and metastasis [54]. Altogether, these results indicated that EVs from tumor-bearing mice upregulated a diverse set of soluble factors in colon fibroblasts to induce the transition of normal cells into CAFs. In addition, the release of cytokines and chemokines in fibroblasts exposed to APC-EVs have oncogenic functions that might be associated with CRC development and progression.

### 3.8. EVs from Tumor-Bearing Mice Promoted an Inflammatory CAF Phenotype via NF-κB Pathway Activation

**Figure 8:**
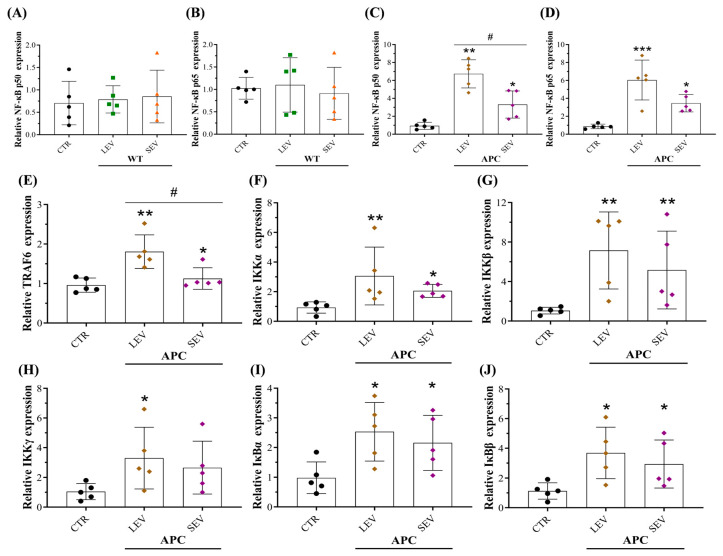
Plasma-derived EVs from tumor-bearing mice promoted fibroblast inflammation by activating the NF-κB pathway. Fibroblasts were treated with WT-derived EVs or APC-derived EVs (70 µg/mL) for 72 h, after which lysates were collected, and qPCR analysis was performed for members and components of the classical NF-κB pathway. Relative expression levels of (**A**,**C**) subunit p50 and (**B**,**D**) subunit p65 in cells exposed to EVs from normal and tumor-bearing mice. Relative expression levels of (**E**) TRAF6, (**F**) IKKα, (**G**) IKKβ, (**H**) IKKγ, (**I**) IκBα, and (**J**) IκBβ in fibroblasts incubated with APC-derived EVs. Data are shown as means ± SD and represent five independent experiments (*n* = 5). Fold changes were determined over normalized cells kept only with a culture medium (CTR). Gene expression was normalized to the housekeeping gene β-actin. * *p* ≤ 0.05, ** *p* ≤ 0.01, *** *p* ≤ 0.001 compared to CTR, # *p* ≤ 0.05 compared to treatment with APC-derived LEVs. One-way ANOVA followed by Dunnett’s multiple comparisons post-test. Abbreviations: NF-κB: Nuclear factor kappa B, TRAF6: Tumor necrosis factor receptor-associated factor 6, IKKα: IκB Kinase alpha, IKKβ: IκB Kinase beta, IKKγ: IκB Kinase gamma, IκBα: nuclear factor of kappa light polypeptide gene enhancer in B-cells inhibitor alpha, IκBβ: nuclear factor of kappa light polypeptide gene enhancer in B-cells inhibitor beta.

Given that the NF-κB signaling pathway has been implicated in transforming the CAF phenotype [55,56,57], we investigated whether both EV subtypes from APC mice would trigger NF-κB activation in colon fibroblasts. We found that CCD-18Co cells incubated with LEV-APC for 72 h exhibited higher mRNA levels of subunits p50 (6.7 times) and p65 (6 times), whereas SEV-APC treatment upregulated the expression levels for both NF-κB subunits equal to 3.3 and 3.4 times, respectively (Figure 8C,D). The expression of NF-κB was not affected in cells by EVs from WT mice (Figure 8A,B). Moreover, components of the classical NF-κB pathway were noticeably increased in colon cells with APC-derived LEV additions, including tumor necrosis factor receptor-associated factor 6 (TRAF6), IKK complex (α, β, and γ), and the cytoplasmic inhibitors of NF-κB (IκB) proteins (Figure 8E–J). Upregulations of total p65, phosphorylated p65, total IκBα, and phosphorylated IκBα in fibroblasts treated with APC-derived EVs were further confirmed at protein levels (Supplementary Figure S12). Therefore, our results proved that EVs derived from tumor-bearing mice reprogramed NFs into an inflammatory CAF phenotype by triggering the activation of the classical NF-κB signaling pathway in colon fibroblasts.

### 3.9. Fibroblasts Educated by APC-Derived EVs Released Soluble Factors with Malignant Behavior Effects on Surrounding Not-Activated Fibroblasts

**Figure 9:**
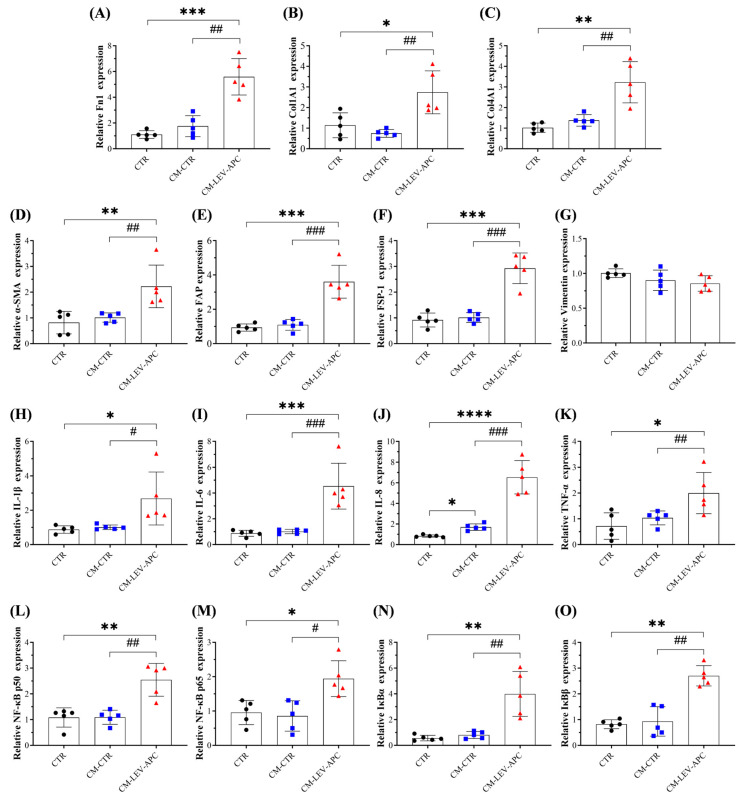
Conditioned media from cells exposed to APC-derived LEVs (CM-LEV-APC) promote fibroblasts’ malignant behavior. Fibroblasts were co-cultured with CM-CTR or CM-LEV-APC at a ratio of 1:1 for 72 h. The mRNA expression levels for (**A**–**C**) ECM components, (**D**–**G**) CAFs markers, (**H**–**K**) pro-inflammatory cytokines, and (**L**–**O**) subunits from the NF-κB pathway were analyzed by PCR. Conditioned media (CMs) from untreated cells (CM-CTR) or treated with LEV-APC (CM-LEV-APC) were collected after 72 h of cell culturing. Data are shown as means ± SD and represent five independent experiments (*n* = 5). Fold changes were determined over normalized cells kept only with a culture medium (CTR). Gene expression was normalized to the housekeeping gene β-actin. * *p* ≤ 0.05, ** *p* ≤ 0.01, *** *p* ≤ 0.001, **** *p* ≤ 0.0001 compared to CTR. # *p* ≤ 0.05, ## *p* ≤ 0.01, ### *p* ≤ 0.001. # *p* ≤ 0.05 compared to CM-CTR. One-way ANOVA followed by Dunnett’s multiple comparisons post-test. Abbreviations: CM-CTR = conditioned media collected from fibroblasts incubated with a medium alone, CM-LEV-APC = conditioned media collected from fibroblasts treated with LEV-APC, ECM: extracellular matrix, Fn1: Fibronectin 1, Col1A1: Collagen type I alpha 1 chain, Col4A1: Collagen type IV alpha 1 chain, α-SMA: Alpha smooth muscle actin, FAP: Fibroblast activation protein, FSP-1: Fibroblast-specific protein 1, TNF-α: Tumor necrosis factor alpha, NF-κB: Nuclear factor kappa B, IκBα: nuclear factor of kappa light polypeptide gene enhancer in B-cells inhibitor alpha, IκBβ: nuclear factor of kappa light polypeptide gene enhancer in B-cells inhibitor beta.

CAFs in the TME have been proven to secrete soluble mediators and EVs that play essential roles in cancer progression by modulating angiogenesis, tumor growth, cell migration and invasion, immunosuppression, cancer recurrence, and metastasis [18,55]. Based on our preliminary results showing that tumor-bearing mice released EVs that educate NFs into an inflammatory CAF phenotype, we hypothesized that CAFs reprogrammed by APC-derived EVs release pro-tumorigenic mediators with the ability to activate neighboring healthy fibroblasts to support CRC growth and dissemination. To mimic the crosstalk between CAFs with surrounding cells via EV communication, CCD18-Co cells were treated with LEV-APC or left untreated for 72 h to obtain conditioned media (CMs) from fibroblasts incubated with a medium alone (referred to as CM-CTR), and CMs from cells treated with LEV-APC (referred as, CM-LEV-APC). Then, CMs from both conditions were co-cultured separately in not-activated fibroblasts for three days. We found that healthy cells exposed to CM-LEV-APC significantly increased the mRNA expressions for ECM components (Fn1, Col1A1, Col14A1) (Figure 9A–C). Fibroblast-activation markers induced by CM treatments were also evaluated. CM-CTR did not induce any significative alteration, while the treatment with CM-LEV-APC resulted in a significant increase in mRNA levels for α-SMA (2.2-fold), FAP (3.6-fold), and FSP-1 (2.9-fold) when compared to the untreated cells (Figure 9D–G). No effects were observed in the mRNA levels for vimentin. Moreover, pro-inflammatory cytokines such as IL-1β (2.7-fold), IL-6 (4.5-fold), IL-8 (6.5-fold), and TNF-α (2-fold) were markedly amplified in colon cells with CM-LEV-APC additions, as compared with control cells (Figure 9H–K). In addition, CM-CTR incubation elevated IL-8 mRNA levels 1.7 times, having no effect on the expression of other cytokines. Finally, the mRNA expression levels of core components and cytoplasmic inhibitors of NF-κB were also investigated. Healthy fibroblasts incubated with CM-LEV-APC for 72 h exhibited higher mRNA levels of subunits p50 (2.5-fold), p65 (2-fold), IκBα (4-fold), and IκBβ (2.7-fold), whereas no alteration in NF-κB signaling was observed in the CM-CTR treatment (Figure 9L–O). Interestingly, fibroblasts co-cultured with CM-LEV-APC showed an influence on the expression of mRNA levels analyzed for ECM components, CAF markers, pro-inflammatory cytokines, and the NF-κB signaling pathway when compared with cells exposed to CM-CTR (Figure 9). Altogether, our present findings support the idea that plasma from the APC^Min/+^ CRC mouse model can reprogram fibroblast behavior via EV communication, activating them into an inflammatory CAF phenotype that is able, in turn, to promote malignant behavior effects in healthy surrounding cells.

## 4. Discussion

CAFs and tumor cells within the TME are known to establish a crosstalk essential for tumor growth and progression. One route involved in this interaction occurs via EVs secreted by both cell types, which can modulate the cellular phenotype to support tumorigenesis [15,18,58]. It has been reported that activated fibroblasts or CAFs release EVs to shape tumor stroma to facilitate tumor development, thus contributing to the malignant behavior of cancer cells [15,59,60]. However, the role of different subpopulations of circulating EVs, including large and small EVs, and how they promote fibroblast activation to enhance the pathogenesis of CRC remain unclear. Herein, we addressed whether circulating EV subtypes from the CRC mouse model APC^Min/+^ could activate normal human colon fibroblasts into CAFs to support CRC dissemination. We explored the role of plasma-derived EVs from APC mice because they are an excellent mouse model bearing multiple intestinal neoplasia (Min) used to simulate familial adenomatous polyposis (FAP) and colorectal tumors in humans [61]. 

Here, we used a differential centrifugation protocol to isolate and quantify pellets enriched with increased amounts of LEVs and SEVs secreted in the plasma. NTA and SEM on EV-containing preparations revealed two populations of vesicles with round shape morphologies and sizes ˃200 nm and <150 nm, a range consistent for large EVs and small EVs, respectively, reported by other authors [24,62]. WB analysis confirmed that both subtypes showed common EV markers CD63 and CD81. Because we did not use additional purification methodologies such as size exclusion chromatography, density gradient, or ultrafiltration, we expected our crude EV preparations to be contaminated with other extracellular particles (e.g., lipoproteins) and soluble proteins such as albumin, immunoglobulins, and fibrinogen [42]. Therefore, the operational terms of EV preparation or EV-containing preparations were used to refer to nanoparticles obtained by multiple centrifugation steps containing populations of LEVs or SEVs and non-vesicular entities. Moreover, our results show that APC mice release significantly higher levels of particles and proteins in plasma than EV preparations from WT mice. In line with these results, it has been shown that CRC patients had more EVs than healthy people [56,63]. As such, plasma EVs in patients with CRC could serve as biomarkers for non-invasive CRC screening and disease progression [64,65]. Once we completed the characterization, we explored the EV biological functions in CCD-18Co, non-malignant human colon fibroblasts. We demonstrated that EV subtypes from tumor-bearing mice, but not from healthy mice, reprogrammed normal fibroblasts into a CAF phenotype by increasing pro-inflammatory responses via the activation of the classical NF-κB signaling pathway. To our knowledge, this is the first demonstration of pro-tumorigenic properties mediated by circulating EVs from APC^Min/+^ mice on human colonic fibroblasts.

Some of the major features of the “activated” state of fibroblasts rely on the migration capacities, ECM deposition, expression of activation markers, and the production of growth factors and cytokines involved in cancer dissemination [7,48]. Our data revealed that APC-derived EVs promoted wound repair and cell migration in a dose and time-dependent manner and upregulated the expression of ECM components, such as collagen and fibronectin, in CCD-18Co fibroblasts. In contrast, WT-derived EVs did not affect the expression of the ECM components analyzed. These findings are in accordance with previous studies conducted by Carvalho and colleagues showing that conditioned media (containing EVs and other soluble mediators) from KRAS-silenced colorectal cancer (HTC116) cells increased ECM components, the migration capacity, and activated CCD-18Co normal-like colon fibroblasts into CAF, suggesting that the inhibition of KRAS might be a potential therapeutic approach for CRC disease [66]. Furthermore, EVs from colorectal adenocarcinoma (SW480) cells activate fibroblasts via the MAPK signaling pathway to allow CRC cells to invade the local tissue without undergoing epithelial-to-mesenchymal transition (EMT) [67]. Thus, our results suggest that tumor-bearing derived EVs could induce cell migration and increase fibronectin and collagen production in colon fibroblasts to remodel the ECM and TME to facilitate tumor cell invasion. Evidence has demonstrated that tumor-derived EVs increase ECM deposition by reprogramming the fibroblast phenotype into a CAF-like state [17,46,47]. We found that treatments with APC-derived EVs, but not those with WT-derived EVs, increased both the mRNA and protein levels of CAF markers, including α-SMA, FAP, FSP-1, and vimentin in CCD-18Co fibroblasts. The increased expression levels of SMA and FAP in this cell line treated with TGF-β or with conditioned media from CRC cells have also been reported by other authors [66,67,68,69]. However, the observation that APC-derived EV subtypes promoted the transformation of NFs into CAFs is unique to this study and is the first such documentation, to the best of our knowledge. 

Pro-inflammatory mediators have been demonstrated to activate fibroblasts into a CAF-like state [56,70]. We showed that fibroblasts activated through tumor-bearing derived EVs showed upregulated expression levels of multiple inflammatory cytokines, such as IL-1β, IL-6, IL-8, TNF-α, and TGF-β, in a time-dependent manner. This effect was not seen in cells exposed to EVs from healthy mice. To confirm the inflammatory effects mediated by APC-EV subtypes, we treated fibroblasts with plasma-WT, plasma-APC, or EV depleted from the plasma of WT or APC mice (referred to as EVFPs). We found that only plasma from APC mice elevated inflammatory cytokines. In parallel, treatments in cells with EVFP-APC (67% particle reduction), composed mainly of soluble proteins, failed to increase cytokine expressions. These findings support the idea that phenotypic transformation in colon fibroblasts was associated with the presence of heterogeneous populations of LEVs and SEVs released in the plasma from tumor-bearing mice. In line with our results, Arebro et al. reported that oral cancer-derived EVs induced an inflammatory CAF phenotype by upregulating multiple pathways involved with inflammation, such as the TNF-α and JAK-STAT signaling pathway [47]. Moreover, TGF-β1-stimulated mesothelial EVs increased ECM production and promoted activation in human peritoneal fibroblasts, and this effect was attenuated after blocking mesothelial EV synthesis/secretion with GW4869 [71]. 

Analysis of the human cytokine antibody array on CCD-18Co cells revealed 20 upregulated cytokines (out of 79 detected cytokines) in the supernatant of LEV-APC activated fibroblasts compared to the supernatant from fibroblasts that received only cell media or WT-derived LEVs, with ENA78, GRO, GRO-Alpha, CCL5, CCL7, and IGF-1 being the cytokines most upregulated (>5.5 times). Clinical conditions in CRC patients with high expression levels of those cytokines and/or pro-inflammatory cytokines such as IL-6 and IL-8 have been associated with drug resistance, distant metastasis, cancer recurrence, and a poor 5-year survival rate [72,73]. Altogether, our data suggests that colon fibroblasts activated by tumor-bearing EVs released cytokines with oncogenic activities that might remodel the tumor microenvironment to initiate CRC growth and dissemination. The excessive secretion of pro-inflammatory factors and oncoproteins in components of the TME sustains the activation of the nuclear factor kappa-light-chain-enhancer of activated B cells (NF-κB), which plays critical roles in all the steps of tumor development from tumor initiation and promotion, invasion, metastasis, anticancer immunity and resistance to therapy [55,74]. We analyzed the activation of NF-κB to gain more insights regarding the pro-tumorigenic properties mediated by circulating EVs released from tumor-bearing mice. Our data revealed that APC-derived EV subtypes, but not EVs from WT mice, upregulated both the mRNA and protein levels of core components from the NF-κB signaling pathway, including inhibitors of NF-κB (IκB) proteins and the IκB kinase (IKK) complex. These results could explain, at least in part, the reason for the high levels of inflammatory cytokines observed in activated fibroblasts exposed to APC-derived EVs. Studies conducted by Wu et al. reported that the nasopharyngeal carcinoma cell line secreted EVs loading the Epstein-Barr virus, EBV-encoded latent membrane protein 1 (LMP1) with the ability to educate primary fibroblasts into CAFs via the NF-κB pathway, and this EV-mediated modulatory activity was reverted when the authors knocked-down the p65 subunit [75]. In addition, EVs secreted by ovarian cancer (OC) cell lines carrying miRNAs promoted CAF activation and OC metastasis via the miR-630/KLF6/NF-κB axis [76]. Collectively, our results demonstrate that APC-EVs might induce pro-tumorigenic activities and CAF-like state transformation in colon fibroblasts by triggering the classical NF-κB pathway. 

Regarding the biological functions mediated by APC-EVs on fibroblast, some minor differences were observed between large and small EVs. For instance, SEVs only elevated the mRNA levels of collagen type I, while LEVs significantly increased all the ECM components analyzed in the study. Furthermore, LEVs upregulated components of the NF-κB more strongly than SEVs. One could explain that tumor-bearing EV subtypes could use different molecular mechanisms to reprogram NFs into CAFs. It is worth noting that our preparations enriched with LEVs or SEVs comprised a heterogeneous population of EVs and other non-EV constituents, which, in combination, reprogrammed the fibroblast phenotype. Therefore, it is possible that the presence of soluble factors co-isolated with the EVs could limit or enhance their biological function in recipient cells. Given that we did not evaluate the effects of purified EVs separated from other non-EV components, we suggest that the functional activities observed here were mediated by EV-enriched preparations rather than EV-specific activity. 

The other important result of our study showed that conditioned media (CMs, which contain EVs, soluble factors) collected from fibroblasts exposed to LEV-APC upregulated the expression of mRNA for ECM, CAF markers, pro-inflammatory cytokines, and the NF-κB signaling pathway in non-activated fibroblasts. In contrast, CM-CTRs from untreated fibroblasts had no effect on the mRNA levels analyzed. Our data indicate that the secretome released by activated fibroblasts stimulated with EV preparations from tumor-bearing mice promotes malignant behavior effects in healthy neighboring cells through paracrine mechanisms, and it might contribute to the initial step for tumor development and metastasis in CRC. In addition, these findings support the hypotheses, to be verified, that EV subtypes from APC mice circulating in the blood could prepare the pre-metastatic niche by stimulating stroma cells to enhance CRC development. These results are in accordance with previous studies in different types of cancer, showing that tumor cell secretome and EVs reprogram fibroblasts and other stroma cells to acquire cancer-supportive behavior essential for TME remodeling and cancer progression [18,75,76,77,78].

A limitation of the current study is that we did not completely explore the downstream signaling pathways by which APC-derived EVs educated normal fibroblasts into CAFs. However, it is possible that the activation of NF-κB could modulate such transformation. Previous investigations of ovarian and nasopharyngeal cancer have indicated that the inhibition of the NF-κB pathway attenuated pro-tumorigenic effects in fibroblasts and reversed the transformation of NFs into CAFs [75,76]. Therefore, future investigation using NF-κB inhibitors (e.g., IMD-0354) or an RNA interference approach uncovered in our study will validate whether tumor-bearing EV subtypes use this transcriptional factor to induce a CAF-like state in human colon fibroblasts. Moreover, for purely technical problems, due to the material limitations and the large number of experiments performed to evaluate and compare the biological functions between LEVs and SEVs from APC mice, we did not use the EV preparations to explore other signaling pathways and molecular mechanisms involved in the EV-mediated pro-tumorigenic activities in human fibroblasts. Another limitation of the present study is that we did not explore purified EVs’ biological functions and compare the vesicles’ cargo. Although our results showed pro-tumorigenic activities mediated by EV subtypes from tumor-bearing mice, we recognize that the presence of other soluble proteins with the EV-containing preparation could enhance or limit the “real” function of individual EV subpopulations. 

## 5. Conclusions

The disclosed data, overall, support the idea that the CRC mouse model APC^Min/+^ released circulating EV subtypes and other non-vesicular particles with the ability to reprogram and promote pro-tumorigenic activities in human colonic fibroblasts, activating them into a CAF-like state by modulating the classical NF-κB signaling pathway, and then, in turn, these activated CAF derived-secretomes stimulated surrounding normal fibroblasts to acquire a cancer-supportive behavior that might contribute to the initial step for tumor growth development in CRC. Our data provide insight into the interaction between plasma-derived EVs from CRC mouse models and human cells, which can be further explored for designing new CRC diagnosis and prognosis tools.

## Figures and Tables

**Table 1:** Primary and secondary antibodies used for Western blotting.

Antibodies	Origin	Dilution	Supplier	Catalog Number#
CD81	Rabbit	1/1000	Thermo Fisher	10630D
CD63	Rabbit	1/1000	Cell signaling (Danvers, MA, USA)	52090
α-SMA	Rabbit	1/1000	Cell signaling	19245
FAP	Rabbit	1/1000	Cell signaling	66562
Vimentin	Rabbit	1/1000	Cell signaling	5741
GAPDH	Rabbit	1/1000	Cell signaling	5174
Actin	Mouse	1/5000	MP Biomedicals (Irvine, CA, USA)	691002
NF-κB p65	Rabbit	1/1000	Cell signaling	8242
Phospho-NF-κB p65	Rabbit	1/1000	Cell signaling	3033
IκBα	Rabbit	1/1000	Cell signaling	4812
Phospho-IκBα	Rabbit	1/1000	Cell signaling	2859
Goat anti-Rabbit IgG	Goat	1/10,000	LI-COR	926-68070
Donkey anti-Mouse IgG	Donkey	1/10,000	LI-COR	926-32212

**Table 2:** Primer sequences used for quantitative real-time PCR.

GENE	Forward	Reverse
*ACTIN*	TGGTTACAGGAAGTCCCTTGCC	ATGCTATCACCTCCCCTGTGTG
*α-SMA*	CCGACCGAATGCAGAAGGA	ACAGAGTATTTGCGCTCCGAA
*FAP*	GGAAGTGCCTGTTCCAGCAATG	TGTCTGCCAGTCTTCCCTGAAG
*FSP-1*	CCTGGGGAAAAGGACAGATGAA	CATGGCAATGCAGGACAGGA
*Vimentin*	AGGCAAAGCAGGAGTCCACTGA	ATCTGGCGTTCCAGGGACTCAT
*IL-1β*	AGCTACGAATCTCCGACCAC	CGTTATCCCATGTGTCGAAGAA
*IL-6*	GGTACATCCTCGACGGCATCT	GTGCCTCTTTGCTGCTTTCAC
*IL-8*	ATGACTTCCAAGCTGGCC	TCTTCAAAAACTTCTCCACAACCC
*TNF-α*	GTGAGGAGGACGAACATC	GAGCCAGAAGAGGTTGAG
*TGF-β*	ACACCAACTATTGCTTCAG	TGTCCAGGCTCCAAATG
*NF-κB p50*	ACACTGGAAGCACGAATGACAGA	CCTCCACCTTCTGCTTGCAA
*NF-κB p65*	CAGGCGAGAGGAGCACAGATAC	TCCTTTCCTACAAGCTCGTGGG
*IKKα*	TGACAGCACAGAGATGGTGA	CTTCTGCTTACAGCCCAACA
*IKKβ*	ACAGCGAGCAAACCGAGTTTGG	CCTCTGTAAGTCCACAATGTCGG
*IKKγ*	AGCACCTGAAGAGATGCCAGCA	AGCCTGGCATTCCTTAGTGGCA
*IκB-α*	CACTCCATCCTGAAGGCTACCAAC	CACACTTCAACAGGAGTGACACCAG
*IκB-β*	GTACTCCCGACACCAACCAT	CGGACCATCTCCACATCTTT
*TRAF6*	CTTTGGCAAATGTCATCTGTG	CTGAATGTGCATGGAATTGG
*Fn1*	AGGCTTGAACCAACCTACGGATGA	GCCTAAGCACTGGCACAACAGTTT
*Col1a1*	TCTGCGACAACGGCAAGGTG	GACGCCGGTGGTTTCTTGGT
*Col4a1*	CTGCCTGGAGGAGTTTAGAAG	GAACATCTCGCTCCTCTCTATG

**Table 3:** Cytokines and chemokines upregulated in human colon fibroblasts treated with pellets enriched with LEVs from tumor-bearing mice.

Cytokines/Chemokines	LEV-APC/Control Fibroblasts (Fold Change)	*p*-Value (LEV-APC/Fibroblasts)
Fold change > 1.5		
IL-6	2.45	0.025
IL-8 ^#^	1.81	0.012
CCL11	3.56	0.047
CCL22 ^#^	2.78	0.029
EGF	2.78	0.046
Angiogenin	3.57	0.021
OSM ^#^	2.12	0.043
VEGF-A	2.06	0.043
FGF-4	2.09	0.042
FLT-3 ligand ^#^	2.20	0.047
IGFBP-3 ^#^	2.80	0.004
CXCL-10	2.63	0.025
Osteopontin ^#^	2.00	0.018
TIMP-2	3.14	0.018
Fold change > 5.5		
GRO-Alpha	6.12	0.0451
CCL5	11.2	0.0058
IGF-1 ^#^	5.93	0.009
Fold change > 20		
CXCL-5 (ENA-78)	29.7	0.011
GRO ^#^	21.8	0.0003
CCL7	23.5	0.0285

^#^ Significance difference when compared to cells treated with WT-derived LEV. Abbreviations: IL-6: Interleukin-6, IL-8: Interleukin-8, ENA78: Epithelial Neutrophil-Activating Peptide 78, GRO: The CXC chemokines growth-regulated oncogene (GRO) alpha, GRObeta, GROgamma, CXCL1: chemokine (C-X-C motif) ligand 1, CCL7:C-C motif chemokine ligand 7, CCL22: C-C motif chemokine 22, CCL5: C-C motif chemokine 5, EGF: Epidermal growth factor, IGF-1:Insulin-like growth factor 1, OSM: Oncostatin M, VEGF-A: Vascular endothelial growth factor A, CCL11:C-C motif chemokine 11, FGF-4: Fibroblast growth factor 4, FLT-3 ligand: FMS-like tyrosine kinase 3 ligand, IGFBP-3: Insulin-like growth factor binding protein 3, CXCL-10: C-X-C motif chemokine ligand 10, TIMP-2: Tissue inhibitor of metalloproteinases 2.

**Table 4:** The relative levels of cytokines expression were compared between LEVs from WT mice and untreated fibroblasts.

Cytokines/Chemokines	LEV-WT/Fibroblasts (Fold Change)	*p*-Value (LEV-WT/Fibroblasts)
IL-6	0.99	0.322
IL-8	1.30	0.320
CCL11	2.30	0.257
CCL22	0.80	0.909
EGF	1.52	0.564
Angiogenin	2.33	0.158
OSM	0.83	0.995
VEGF-A	0.75	0.875
FGF-4	0.89	0.967
FLT-3 ligand	0.70	0.745
IGFBP-3	0.41	0.183
CXCL-10	1.24	0.709
Osteopontin	0.84	0.708
TIMP-2	1.68	0.569
GRO-Alpha	3.383	0.3044
CCL5	2.793	0.5158
IGF-1	2.522	0.2642
CXCL-5	1.26	0.9987
GRO	2.327	0.6406
CCL7	1.135	0.7315

## Data Availability

The authors declare that all data presented in this manuscript are fully available.

## Supplementary Materials

The following supporting information can be downloaded at: https://www.mdpi.com/article/10.3390/cells13141195/s1, Figure S1: Tissue preparation and tumor counting, Figure S2: Physical characterization of plasma-derived EV subtypes form WT and APC^Min/+^ mice, Figure S3: Morphological characterization of EV subtypes from WT and APC^Min/+^ mice, Figure S4: Wound recovery assay in human colonic fibroblasts, Figure S5: Depletion of particle concentration in plasma from WT and APC^Min/+^ mice, Figure S6: Plasma from WT and APC increased wound recovery in fibroblasts, Figure S7: Plasma-derived EVs from tumor-bearing mice increased cancer-associated fibroblast (CAF) protein levels in fibroblasts, Figure S8: Cancer-associated fibroblast (CAF) markers, Figure S9: Pro-inflammatory cytokines levels in fibroblasts treated with plasma-derived EVs from WT mice, Figure S10: Pro-inflammatory cytokines levels in fibroblasts treated with plasma-derived EVs from WT mice, Figure S11: Cytokine antibody arrays expression levels, Figure S12: Plasma-derived EVs from tumor-bearing mice increased NF-κB protein levels.
