# Supplementary material for: Plasma-Derived Extracellular Vesicles and Non-Extracellular Vesicle Components from APCMin/+ Mice Promote Pro-Tumorigenic Activities and Activate Human Colonic Fibroblasts via the NF-κB Signaling Pathway
Source: Cells. 2024 Jul 15;13(14):1195. doi: 10.3390/cells13141195 (PMC11274984; doi:10.3390/cells13141195)
Supplement: Supplementary file 1 [file cells-13-01195-s001.zip › cells-3081886-supplementary.pdf]

Supplementary Figure S1. Tissue preparation and tumor counting. Small intestine and colon from **(A)** WT and **(B)** APC$^{Min/+}$ mice. **(C)** SI and Co tumor count from APC mice (n=11). 15-week-old males and females WT and APC were used to collect the SI and Co. The intestinal tract was surgically removed and sectioned into the small intestine and colon. Tissue samples were fixed overnight in 10% buffered formalin. The sections were stained with 0.1 methylene blue for 1 min. Tumors in each section were counted under a dissecting scope. Abbreviations: SI: small intestine, Co: colon.

Supplementary Figure S2. Physical characterization of Plasma-derived EV subtypes from WT and APC$^{Min/+}$ mice. NTA analysis showing the mean and mode size of pellets enriched with LEV and SEV from **(A)** WT and **(B)** APC$^{Min/+}$ mice (n=13). (**C-D**) The percentage of particles with sizes below 150 nm or over 151 nm recovered with the differential centrifugation protocol proposed in the study. Measurements were performed in individual pellets enriched with LEV or SEV from (C)WT or (D) APC mice (n=13). The particle yield of LEV was calculated by summing the number of particles with a size over 151 nm and then dividing by the average of the total number of particles present in the pellet. The same strategy was used to calculate the particle yield of the pellet enriched with SEV.

Supplementary Figure S3. Morphological characterization of EV subtypes from WT and APC$^{Min/+}$ mice. SEM microscopy images showing homogeneous round-shaped vesicles and other particles in pellets enriched with **(A)** LEV or **(B)** SEV from WT and APC mice. Particle sizes range from 50–600 nm. Scale bars = 400 nm and 200 nm for LEV and SEV, respectively. (**C-D**) SEM analysis showing the (C) mean and (D) mode size of pellets enriched with LEV and SEV from WT and APC mice (number of images analyzed per mouse=5). (**E-F**) The percentage of particles with sizes below 150 nm or over 151 nm was recovered with differential centrifugation and then analyzed by SEM.

Supplementary Figure S4. Wound recovery assay in human colonic fibroblasts. Wound closure percentages of CCD-18Co fibroblasts treated with LEV or SEV from **(A, B)** WT and **(D, E)** APC mice at indicated time points and concentration of EVs. **(C, F)** represent the wound recovery of fibroblasts exposed with recombinant human TGF-β1 (10 ng/mL) analyzed in the experiments for WT-derived EVs or APC-derived EVs. Data are shown as means ± SD and represent four independent experiments with two technical replicates (n=4). Fold changes were determined over normalized cells kept only with culture medium (CTR). *P ≤ 0.05, **P ≤ 0.01, ***P ≤ 0.001. One-way ANOVA followed by Dunnett's multiple comparisons post-test.

Supplementary Figure S5. Depletion of particle concentration in plasma from WT and APC$^{Min/+}$ mice. The supernatants (non-pellet) from 150,000 x g centrifugation were subjected to final ultracentrifugation at 150,000 x g for 20h and filtered through a 0.22 µm. Fold changes were calculated by NTA analysis over particles present in the plasma of (A)WT or (B) APC mice without any centrifugation steps. ****P ≤ 0.0001. One-way ANOVA followed by Dunnett's multiple comparisons post-test.

Supplementary Figure S6. Plasma from WT and APC increased wound recovery in fibroblasts. (A) Fibroblast wound healing was stimulated with the supernatant of plasma-WT or plasma-APC (70 μg/mL) at indicated time points. **(B)** When indicated, a supernatant of plasma-WT or plasma-APC depleted with EVs (referred to as EVFP-WT or EVFP-APC) was added to fibroblasts at the same concentration as plasma samples. Dotted lines represent the size of the original wound (Bars=200 μm). Recombinant human TGF-β1 (10 ng/mL) was used as a positive control. Graphs show the percentage of the wounded area in fibroblasts treated with **(C)** plasma or **(D)** EVFP at indicated time points with respect to the original wound. Data are shown as means ± SD and represent four independent experiments with two technical replicates (n=4). Fold changes were determined over normalized cells kept only with culture medium (CTR). $^{**}P \leq 0.01$, $^{***}P \leq 0.001$, $^{****}P \leq 0.0001$. One-way ANOVA followed by Dunnett's multiple comparisons post-test. Abbreviations: EV-free plasma.

Supplementary Figure S7. Plasma-derived EVs from tumor-bearing mice increased cancer-associated fibroblast (CAF) protein levels in fibroblasts. Representative western blot illustrating that (**A-C**) LEV and (**D-F**) SEV from APC mice increased protein levels of α-SMA, FAP, FSP-1, and Vimentin in CCD-18Co cells. The CAF markers were analyzed in fibroblasts 72h post-treatment. Relative band intensity was calculated using ImageJ. Protein levels were normalized to GAPDH levels. Fold changes were calculated over normalized cells kept only with culture medium (CTR). $^{*}P \leq 0.05$, $^{**}P \leq 0.01$, $^{***}P \leq 0.001$. One-way ANOVA followed by Dunnett's multiple comparisons post-test. Abbreviations: CAF: cancer-associated fibroblast, α-SMA: Alpha smooth muscle actin, FAP: Fibroblast activation protein, FSP-1: Fibroblast-specific protein 1.

Supplementary Figure S8. Cancer-associated fibroblast (CAF) markers. Original blots corresponding to Fig S7. The CAF markers, including α-SMA, FAP, FSP-1, and Vimentin, were analyzed in fibroblasts 72h post-treatment with **(A-B)** LEV and **(C)** SEV isolated from WT or APC mice. Protein bands were captured by Odyssey Fc Imaging System (LI-COR). Lanes not included in Supplementary Figure S7 are marked with "X".

Supplementary Figure S9. Pro-inflammatory cytokines levels in fibroblasts treated with plasma and EVFP from tumor-bearing mice. Relative expression levels of **(A, F)** IL-1β, **(B, G)** IL-6, **(C, H)** IL-8, **(D, I)** TNF-α, and **(E, J)** TGF-β in fibroblasts exposed with supernatant of plasma or EVFP (70 μg/mL) from APC mice. The RNA expression levels were evaluated by qPCR in fibroblasts at 72h post-treatment. Data are shown as means ± SD and represent five independent experiments (n=5). Fold changes were determined over normalized cells kept only with culture medium (CTR). Gene expression was normalized to the housekeeping gene β-actin. Abbreviations: TNF-α: Tumor necrosis factor alpha, TGF-β: Transforming growth factor beta, EVFP: EV-free plasma.

Supplementary Figure S10. Pro-inflammatory cytokines levels in fibroblasts treated with plasma-derived EVs from WT mice. Relative expression levels of **(A)** IL-1β, **(B)** IL-6, **(C)** IL-8, **(D)** TNF-α, and **(E)** TGF-β in fibroblast exposed with pellet enriched with LEV and SEV from WT. The RNA expression levels were evaluated by qPCR in fibroblasts at 72h post-treatment. Data are shown as means ± SD and represent five independent experiments (n=5). Fold changes were determined over normalized cells kept only with culture medium (CTR). Gene expression was normalized to the housekeeping gene β-actin. Abbreviations: TNF-α: Tumor necrosis factor alpha, TGF-β: Transforming growth factor beta.

Supplementary Figure S11. Cytokine antibody arrays expression levels. Fold change analysis of cytokines corresponding to Fig. 7. Numbers in red represent the cytokines/chemokines in the antibody arrays. 1= ENA78 (CXCL-5), 2=GRO (alpha/beta/gamma), 3=GRO alpha (CXCL1), 4= IL-6, 5= IL-8, 6= CCL7, 7= CCL22, 8=Rantes (CCL5), 9= EGF, 10= IGF-1, 11= Angiogenin, 12= OSM, 13=VEGF-A, 14= CCL11, 15= FGF-4, 16= FLT-3 ligand, 17= IGFBP-3, 18= CXCL-10, 19= Osteopontin, 20= TIMP-2. Data are shown as means ± SD and represent three independent experiments (n=3). Fold changes of the selected cytokines/chemokines were determined to the average of the positive controls. *P ≤ 0.05, **P ≤ 0.01, ***P ≤ 0.001 compared to CTR, # P ≤ 0.05, ## P ≤ 0.01 compared to treatment with WT-derived LEV. One-way ANOVA followed by Dunnett's multiple comparisons post-test.

Supplementary Figure S12. Plasma-derived EVs from tumor-bearing mice increased NF-κB protein levels. Fibroblasts were treated with APC-derived EVs (70µg/mL) for 72h, after which lysates were collected and WB analysis was performed for components of the NF-κB pathway. Relative expression levels of **(A)** total p65, **(B)** Phospho-p65, **(C)** total IκBα, and **(D)** **P**hospho-IκBα in cells exposed with EVs from normal tumor-bearing mice. **(E)** Original blots corresponding to bar graphs (A-D). Protein bands were captured by Odyssey Fc Imaging System (LI-COR). Protein levels were normalized to GAPDH levels. Relative band intensity was calculated using ImageJ. Fold changes were determined over normalized cells kept only with culture medium (CTR). *P ≤ 0.05, **P ≤ 0.01. One-way ANOVA followed by Dunnett's multiple comparisons post-test. Abbreviations: NF-κB: Nuclear factor kappa B, IκBα: nuclear factor of kappa light polypeptide gene enhancer in B-cells inhibitor alpha.
