# Supplementary figures and images for: Plasma-Derived Extracellular Vesicles and Non-Extracellular Vesicle Components from APCMin/+ Mice Promote Pro-Tumorigenic Activities and Activate Human Colonic Fibroblasts via the NF-κB Signaling Pathway
Source: Cells. 2024 Jul 15;13(14):1195. doi: 10.3390/cells13141195 (PMC11274984; doi:10.3390/cells13141195)

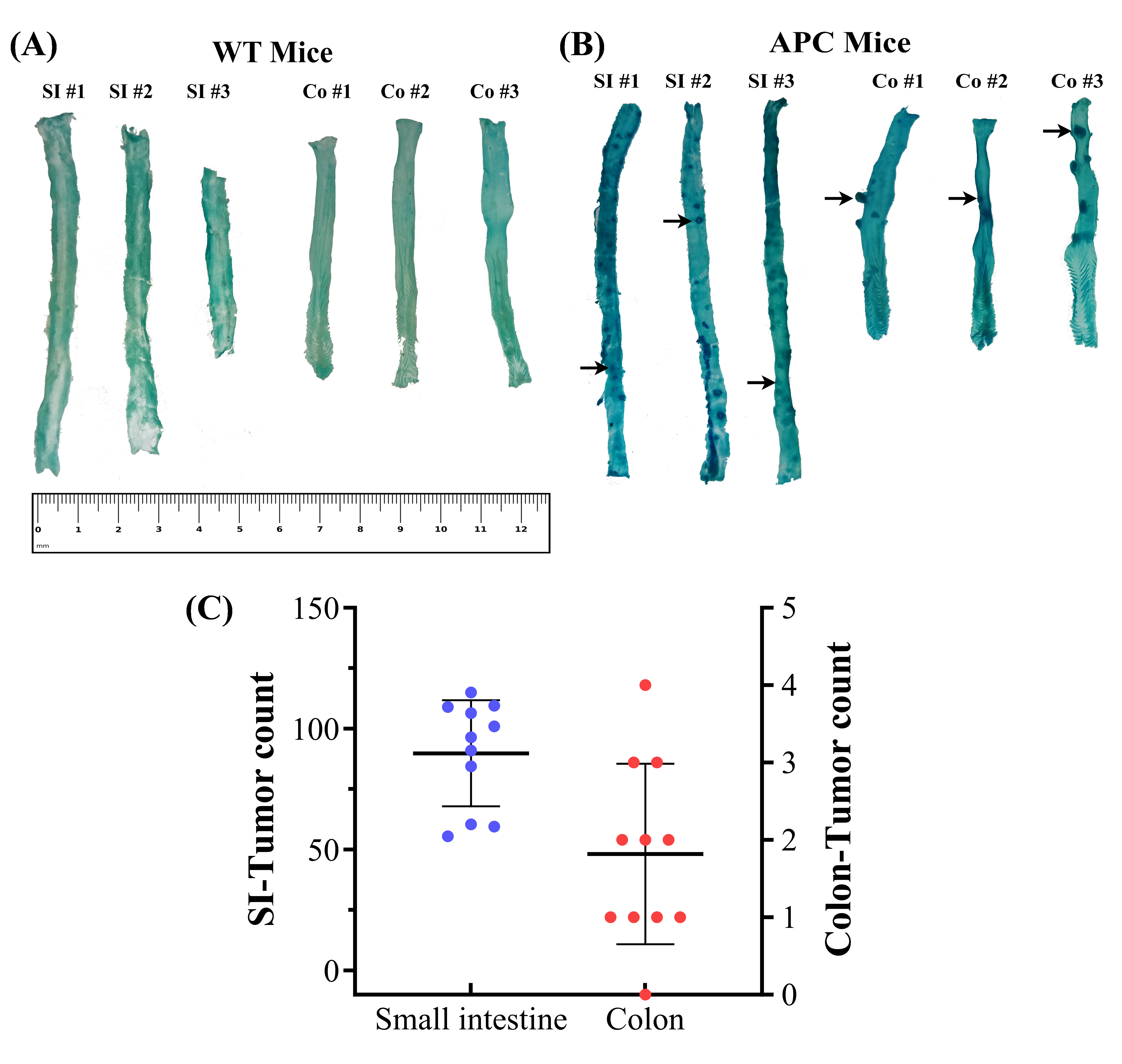

Supplement: Supplementary file 1 [file cells-13-01195-s001.zip › Supplementary Figure S1.png]

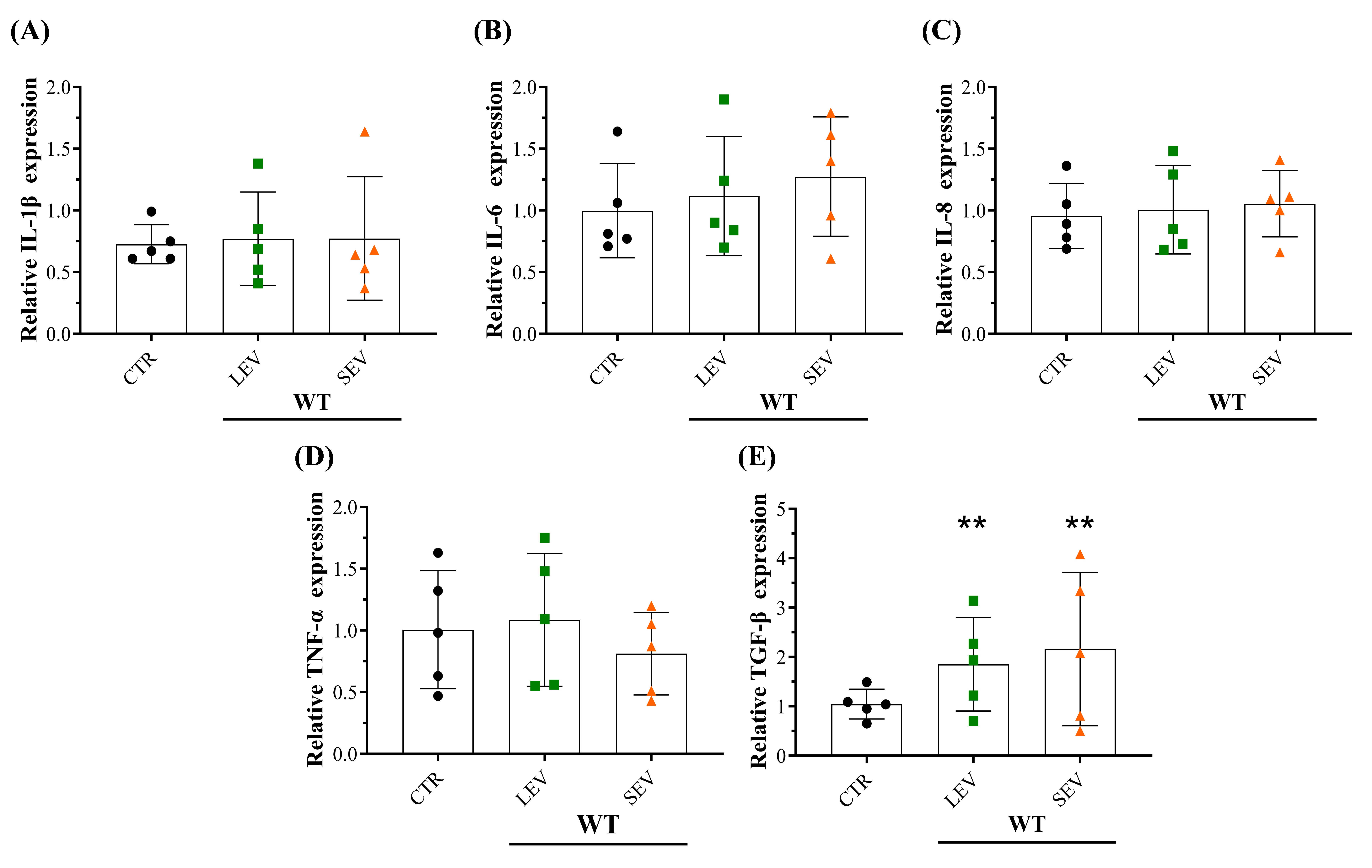

Supplement: Supplementary file 1 [file cells-13-01195-s001.zip › Supplementary Figure S10.png]

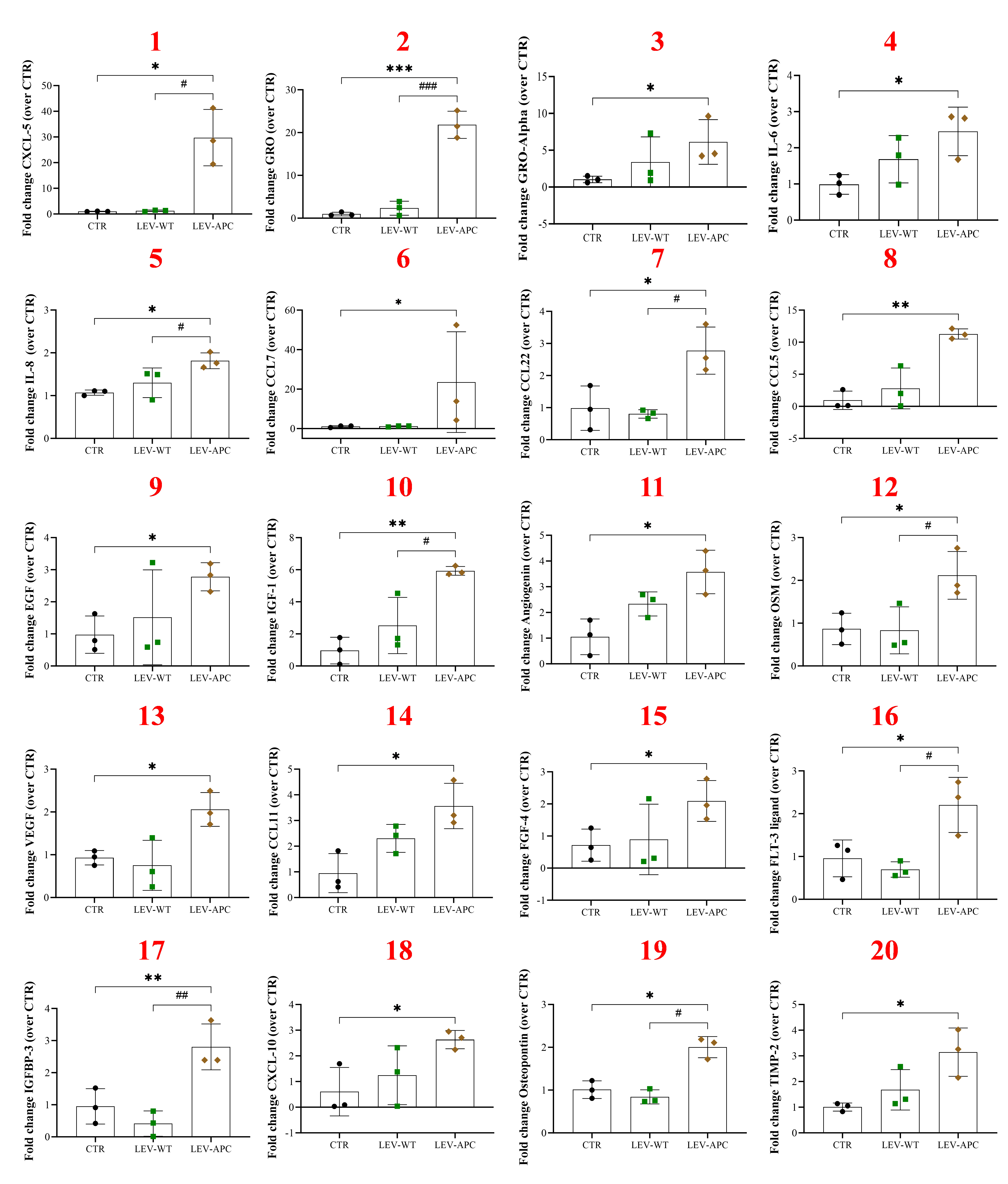

Supplement: Supplementary file 1 [file cells-13-01195-s001.zip › Supplementary Figure S11.png]

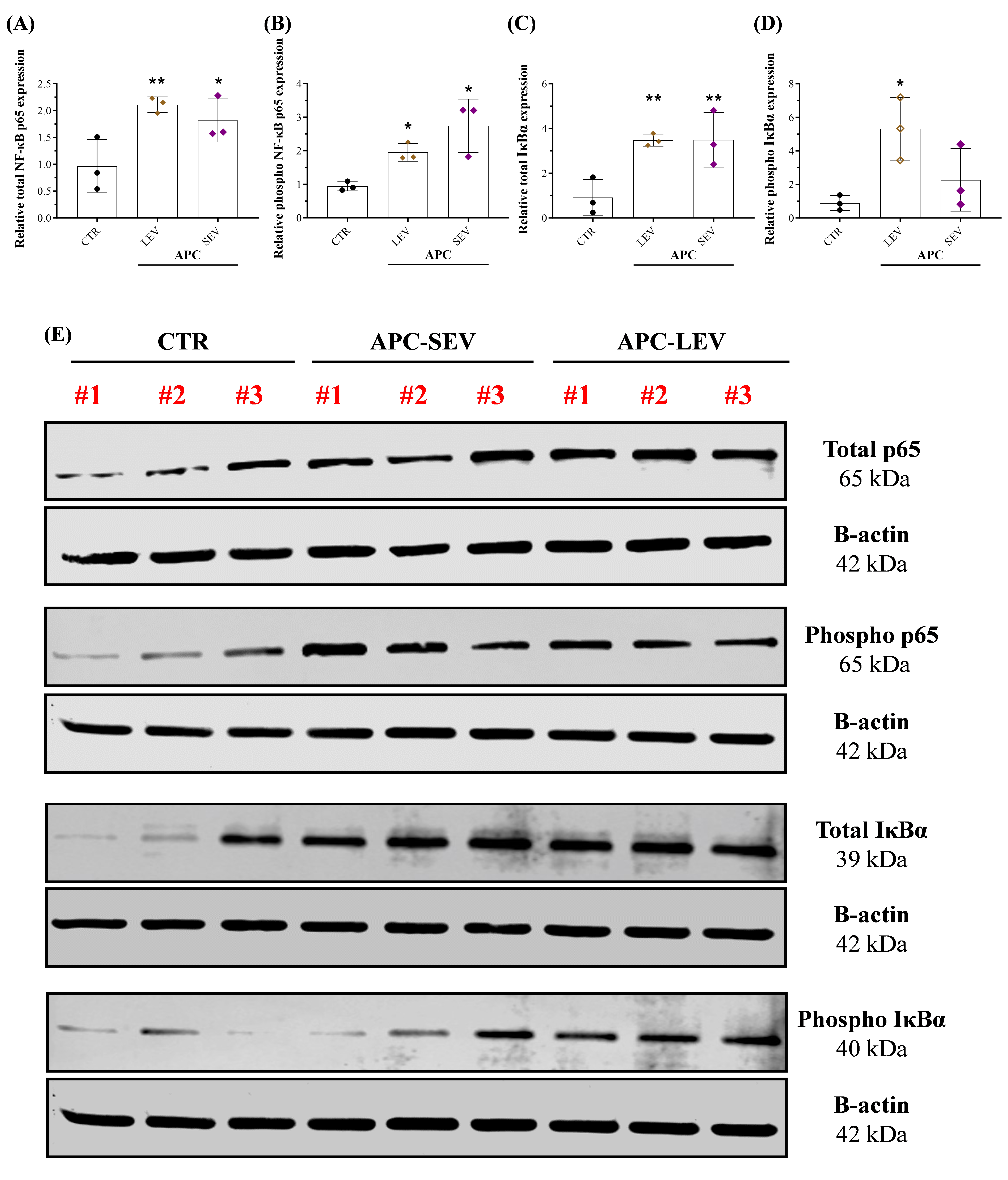

Supplement: Supplementary file 1 [file cells-13-01195-s001.zip › Supplementary Figure S12.png]

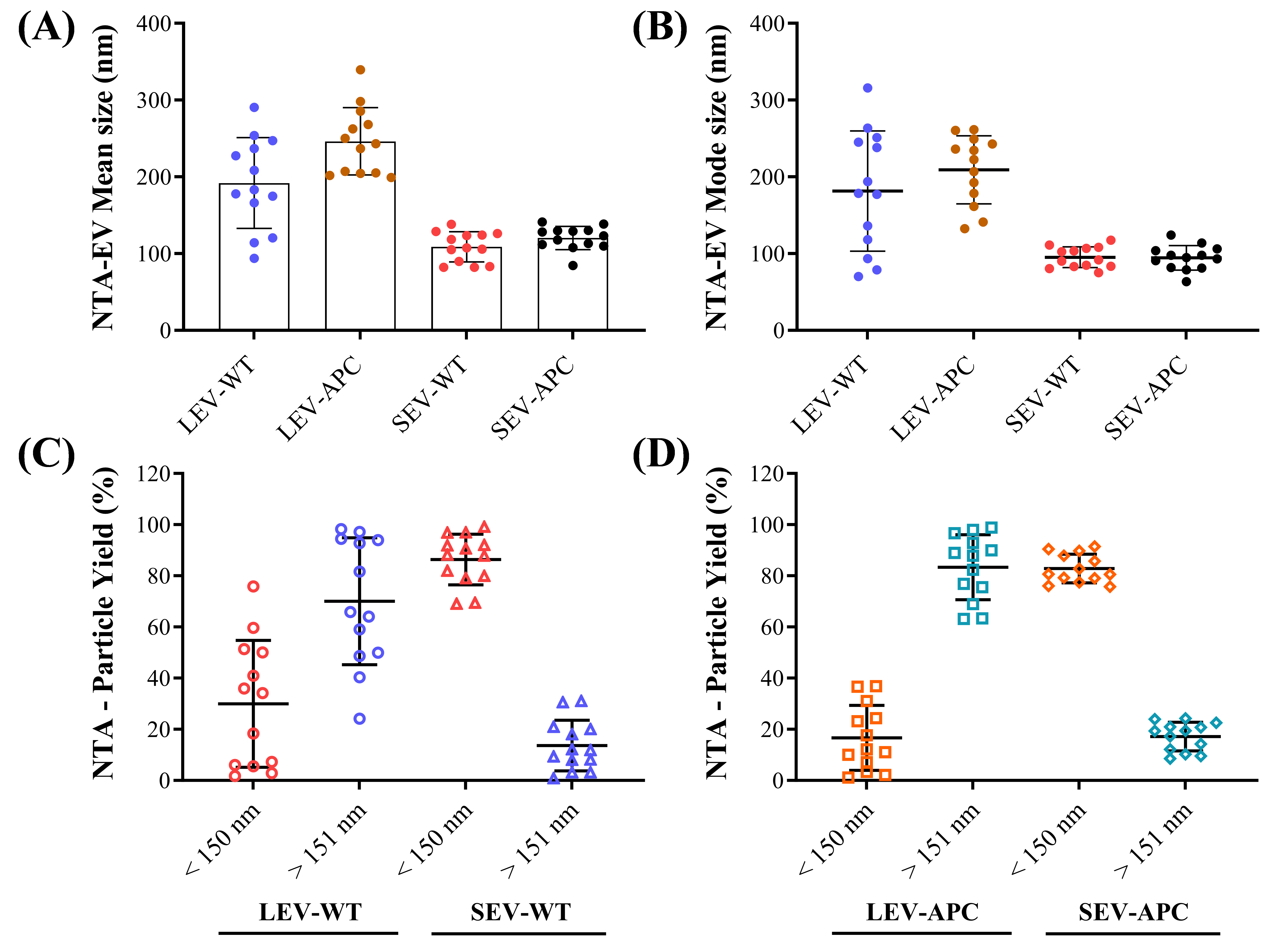

Supplement: Supplementary file 1 [file cells-13-01195-s001.zip › Supplementary Figure S2.png]

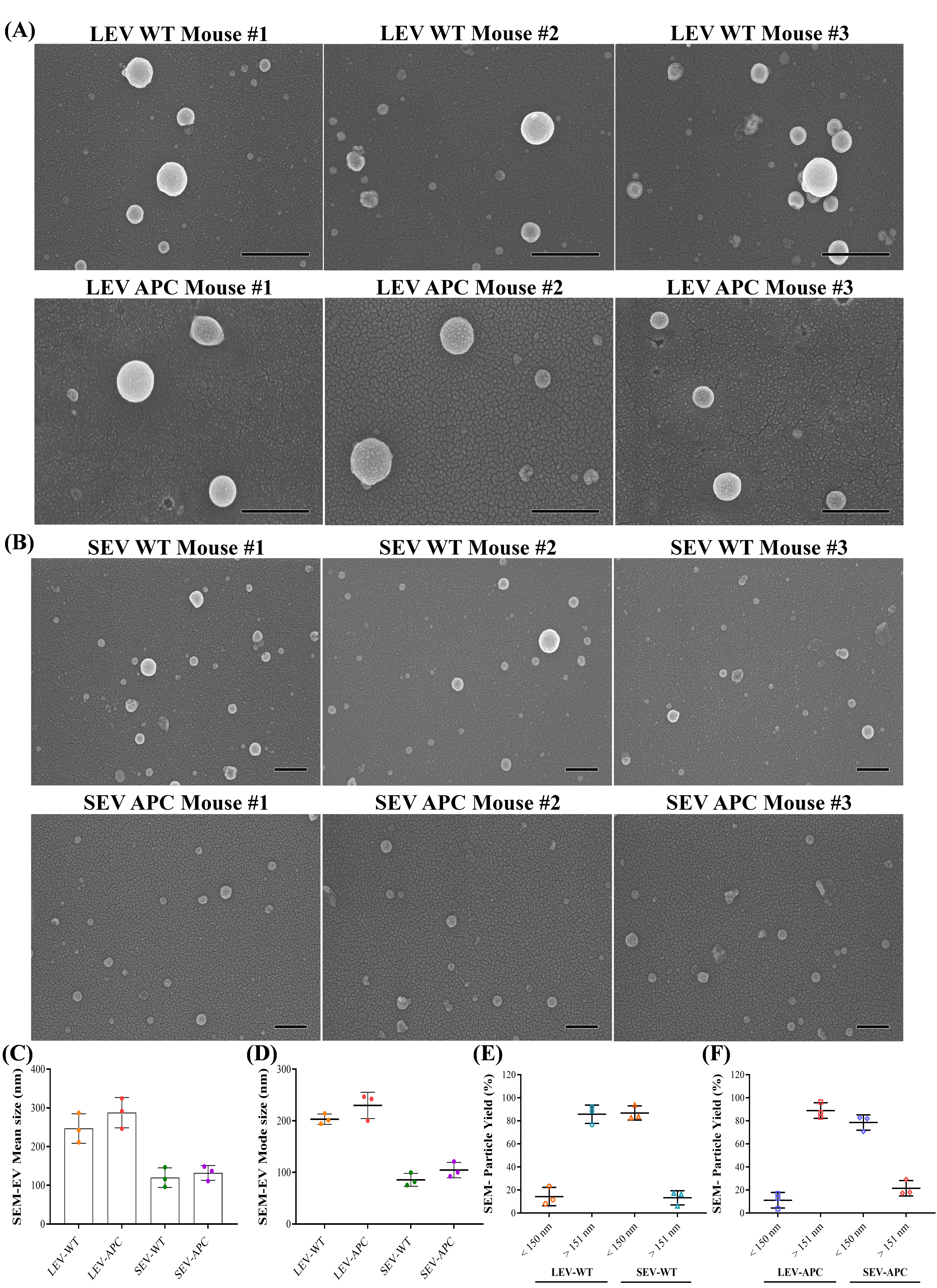

Supplement: Supplementary file 1 [file cells-13-01195-s001.zip › Supplementary Figure S3.png]

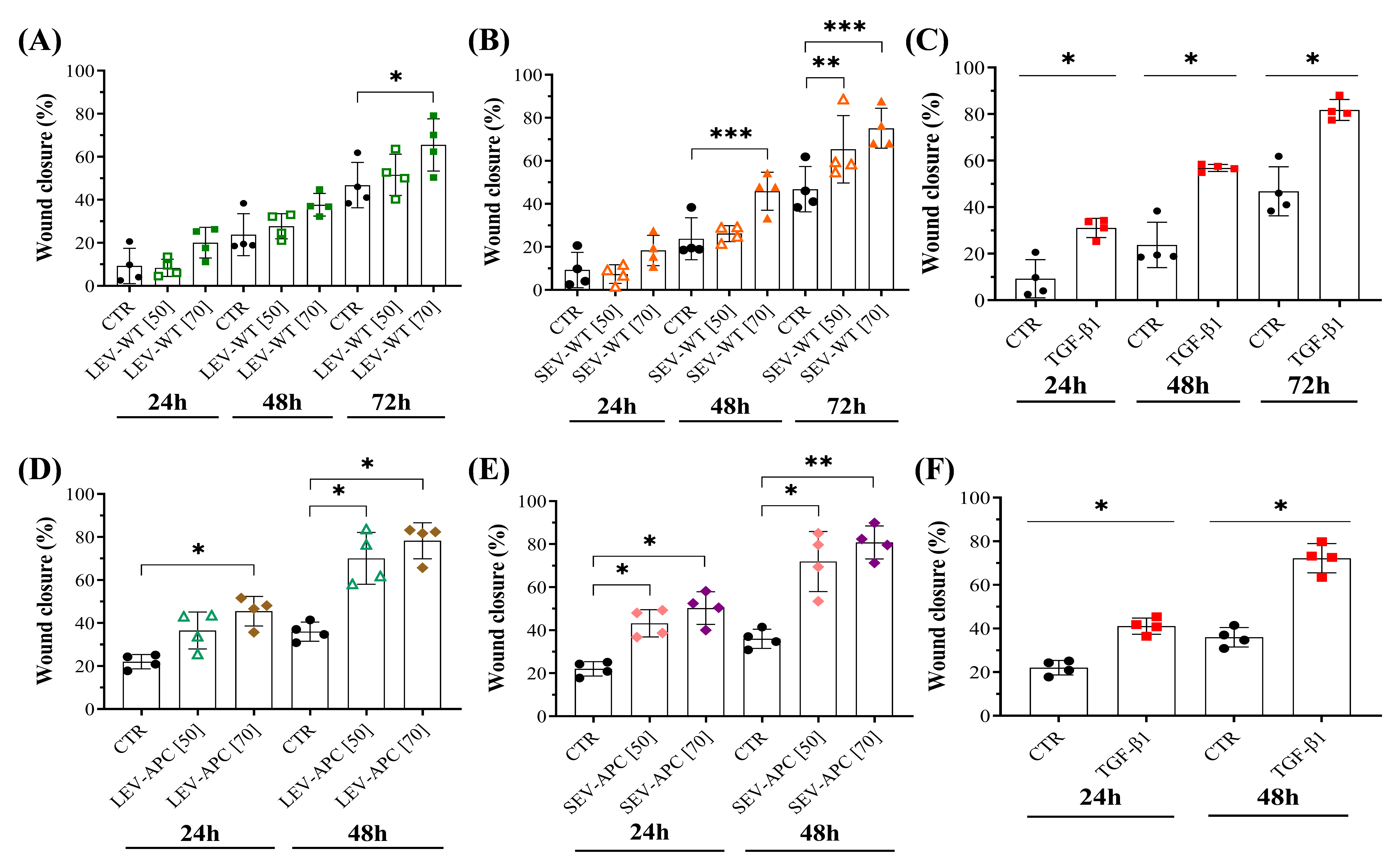

Supplement: Supplementary file 1 [file cells-13-01195-s001.zip › Supplementary Figure S4.png]

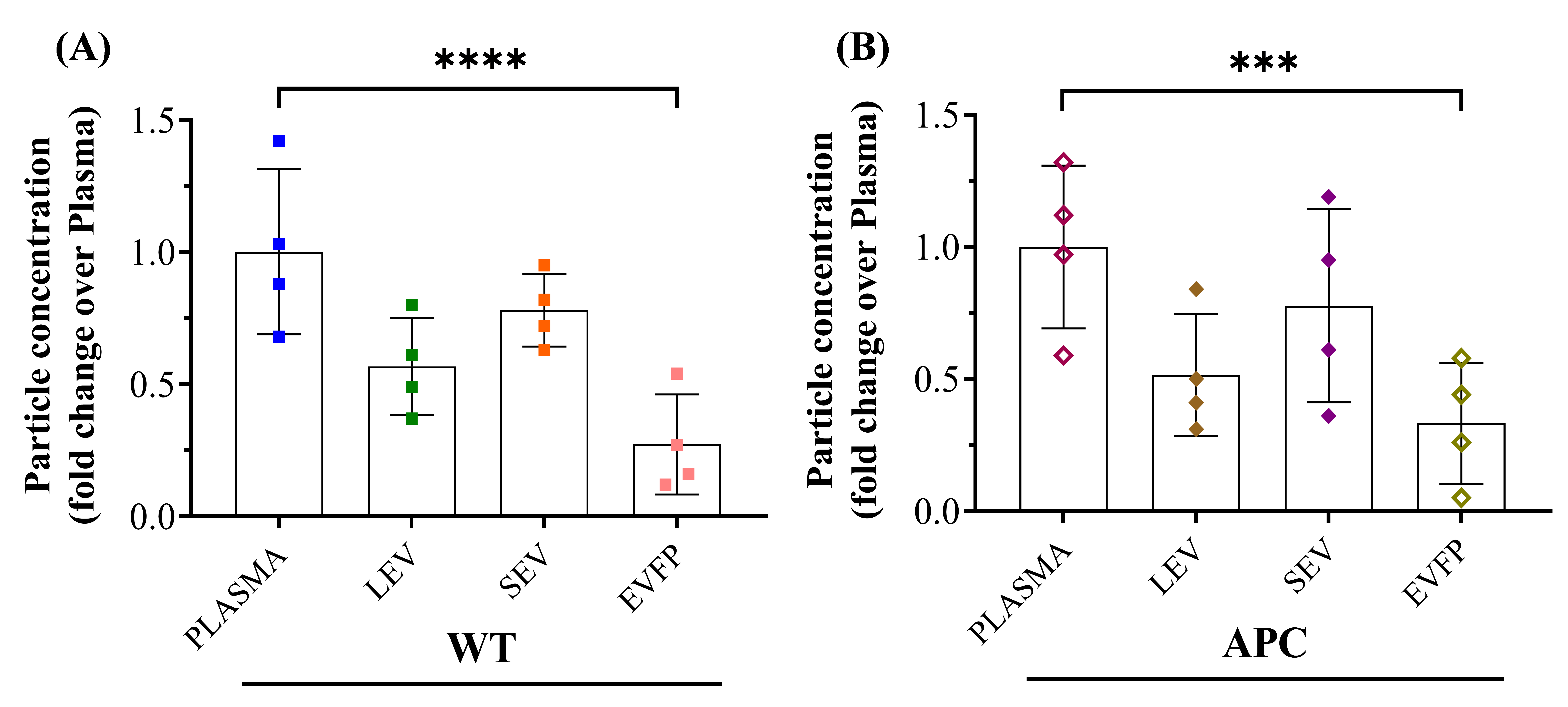

Supplement: Supplementary file 1 [file cells-13-01195-s001.zip › Supplementary Figure S5.png]

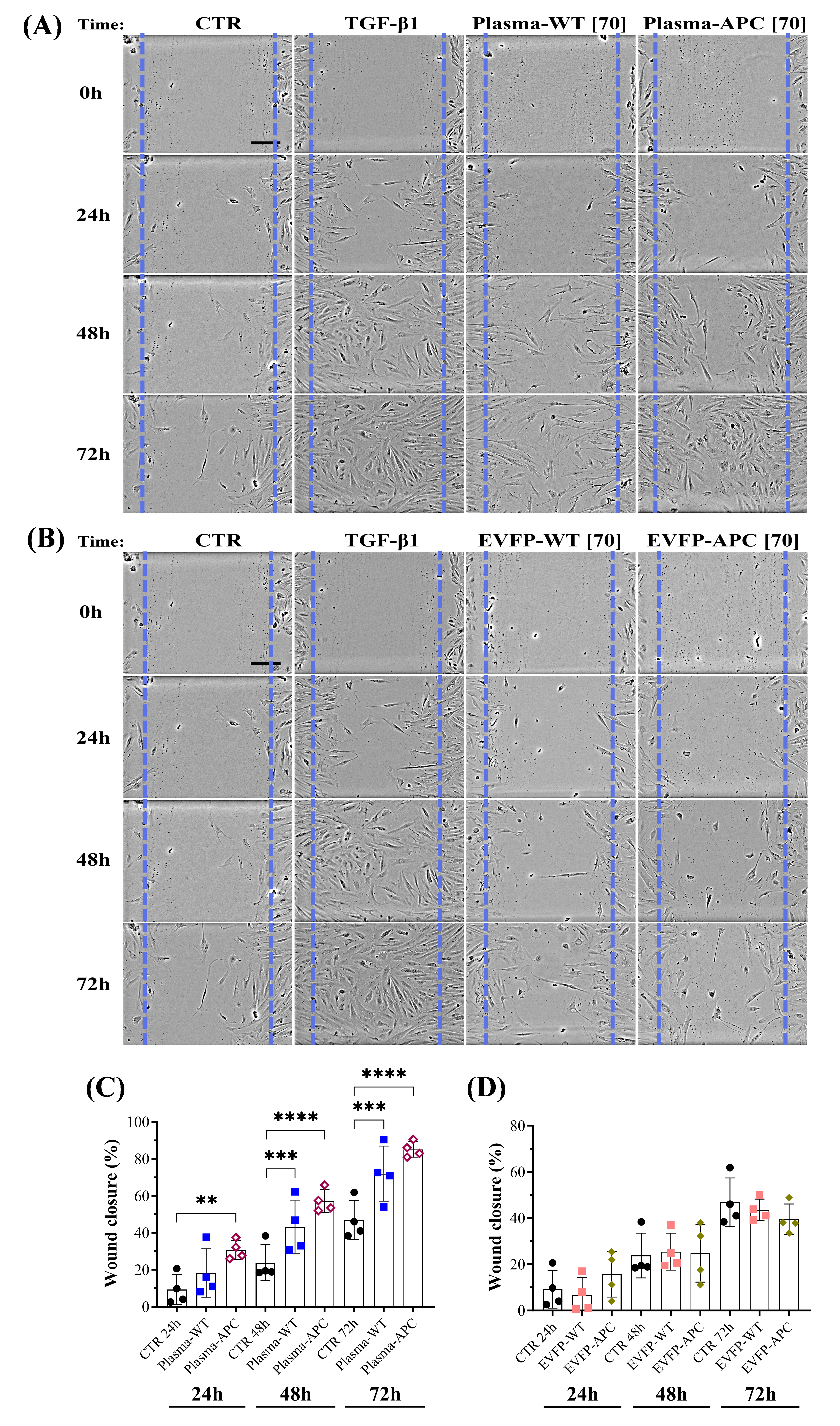

Supplement: Supplementary file 1 [file cells-13-01195-s001.zip › Supplementary Figure S6.png]

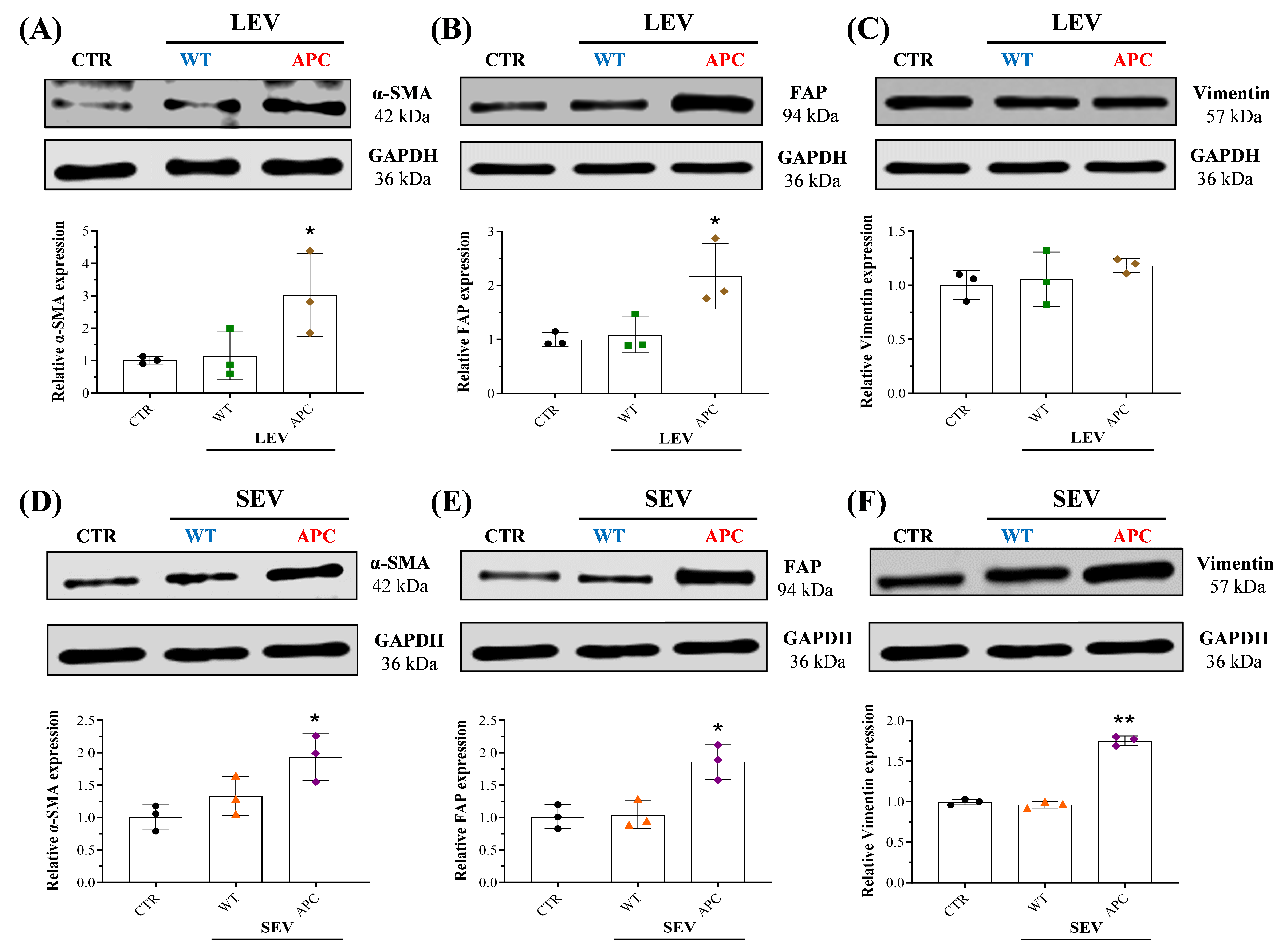

Supplement: Supplementary file 1 [file cells-13-01195-s001.zip › Supplementary Figure S7.png]

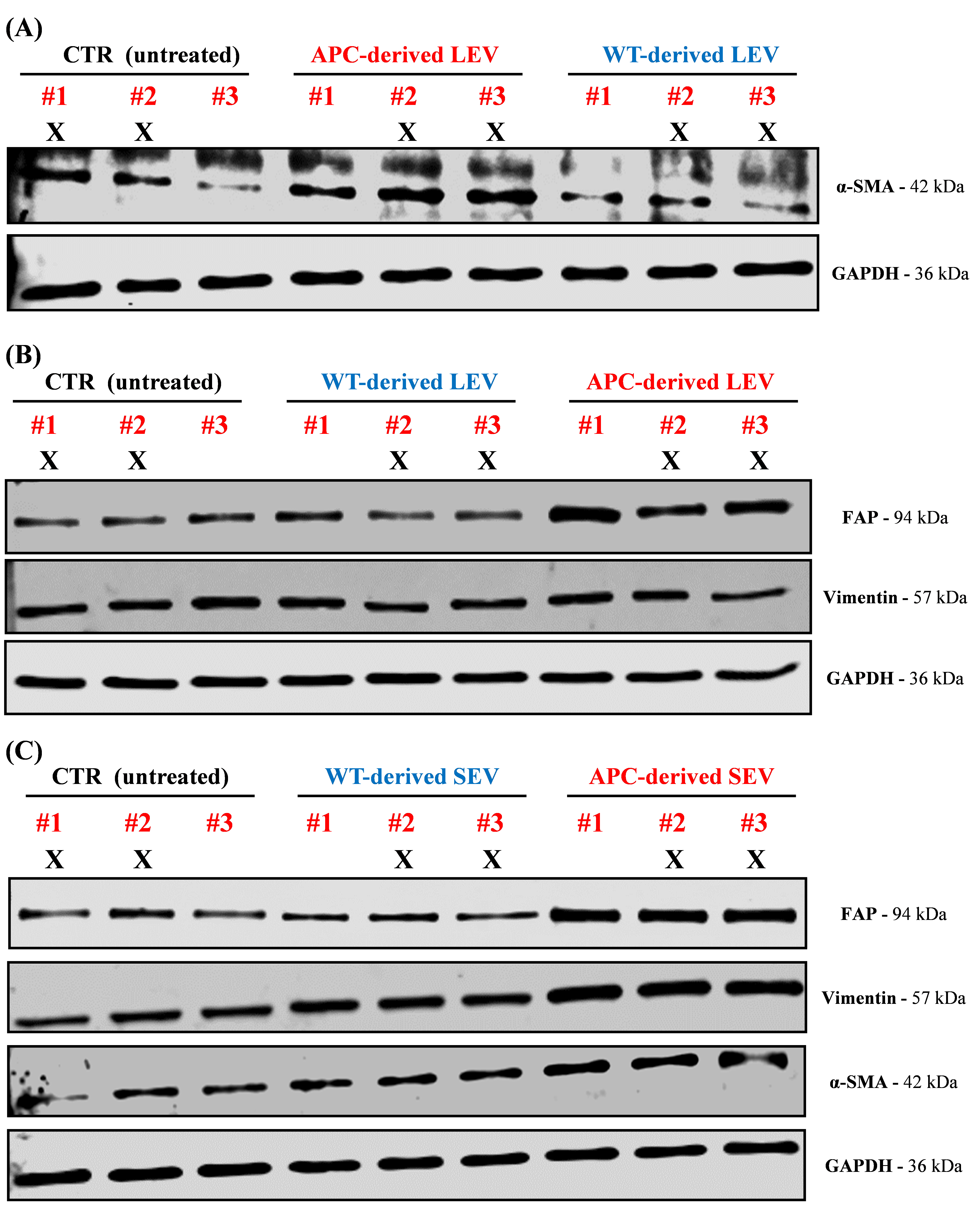

Supplement: Supplementary file 1 [file cells-13-01195-s001.zip › Supplementary Figure S8.png]

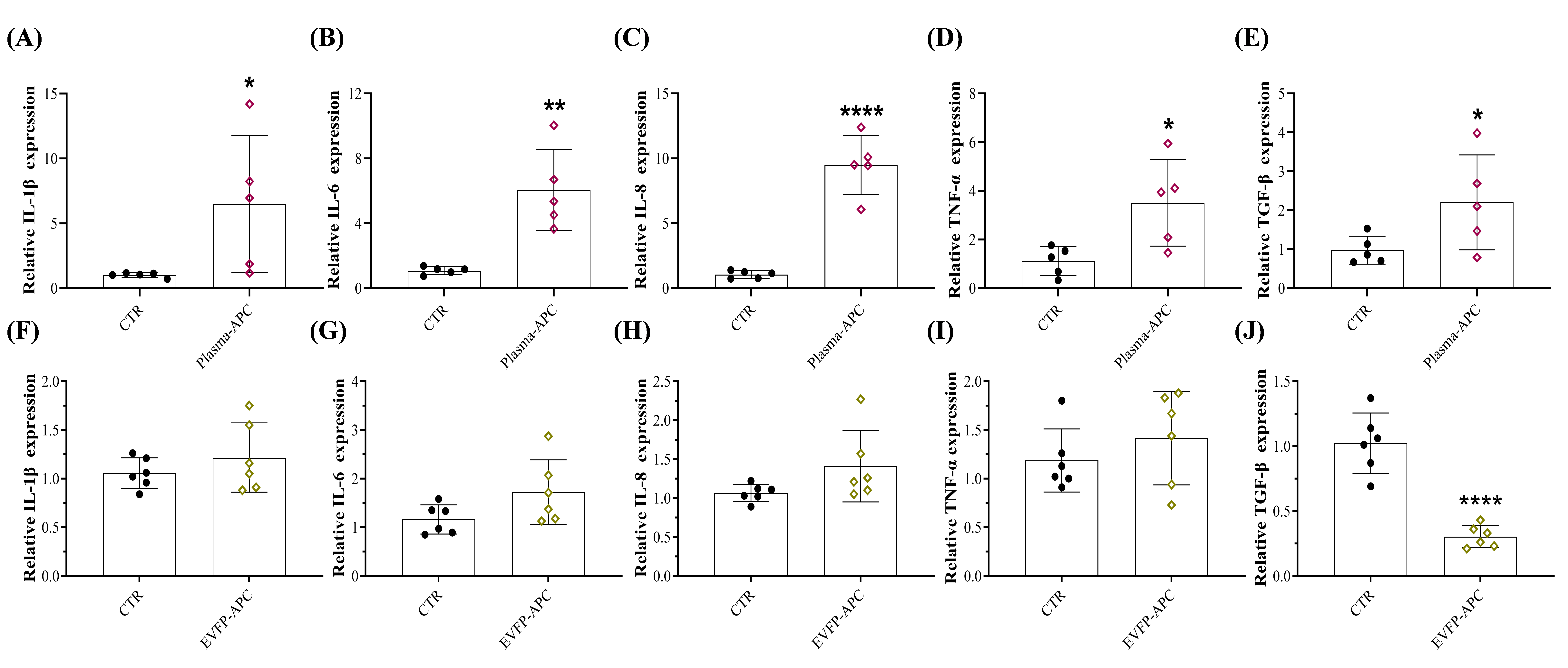

Supplement: Supplementary file 1 [file cells-13-01195-s001.zip › Supplementary Figure S9.png]
